# Social media influences National Park visitation

**DOI:** 10.1073/pnas.2310417121

**Published:** 2024-04-01

**Authors:** Casey J. Wichman

**Affiliations:** ^a^School of Economics, Georgia Institute of Technology, Atlanta, GA 30332; ^b^Resources for the Future, Washington, DC 20036

**Keywords:** National Parks, recreation, social media, environmental preservation, congestion

## Abstract

Visitation to National Parks in the United States increased by more than 25% since 2010, rising from roughly 70 to 90 million annual visitors. Anecdotes suggest that this increase was driven by the advent of social media in the early-to-mid 2010s, generating a new form of exposure for parks, and has led to concerns about overcrowding and degradation of environmental quality. However, there is little empirical evidence on the role of social media in influencing recreation decisions. Here, I construct a dataset on social media exposure (SME) for each National Park and relate that exposure to changes in visitation over the last two decades. High SME parks see visitation increase by 16 to 22% relative to parks with less exposure, which comes with a concomitant increase in revenue. Low SME parks have no, or negative, changes in visitation. These estimates account for unobserved park heterogeneity and are based on an instrumental variables strategy that predicts exposure with a park’s online popularity prior to the social media era. Additional analysis suggests that recent social media posts that include media attachments increase visitation, while posts with negative sentiment reduce visitation. These results provide insight for the National Park Service—which faces more than $22 billion in deferred maintenance costs and is considering policy options to manage demand—as well as for management of recreation on other public lands.

## Significance

Media has long been influential in generating support for environmental preservation, specifically within the US National Park Service. However, the extent to which social media influences recreational visits to National Parks is unclear. Here, I develop a measure of social media exposure and estimate that parks with high exposure see increases in visitation that are 16 to 22% larger than parks with less exposure, that see little change. Social media posts with media attachments and positive sentiment garner larger visitation effects. These findings can aid the National Park Service in understanding the drivers of recreation decisions as they struggle with concerns about deferred maintenance costs and implement strategies to manage the increased demand for parks.

**Fig. 1.**
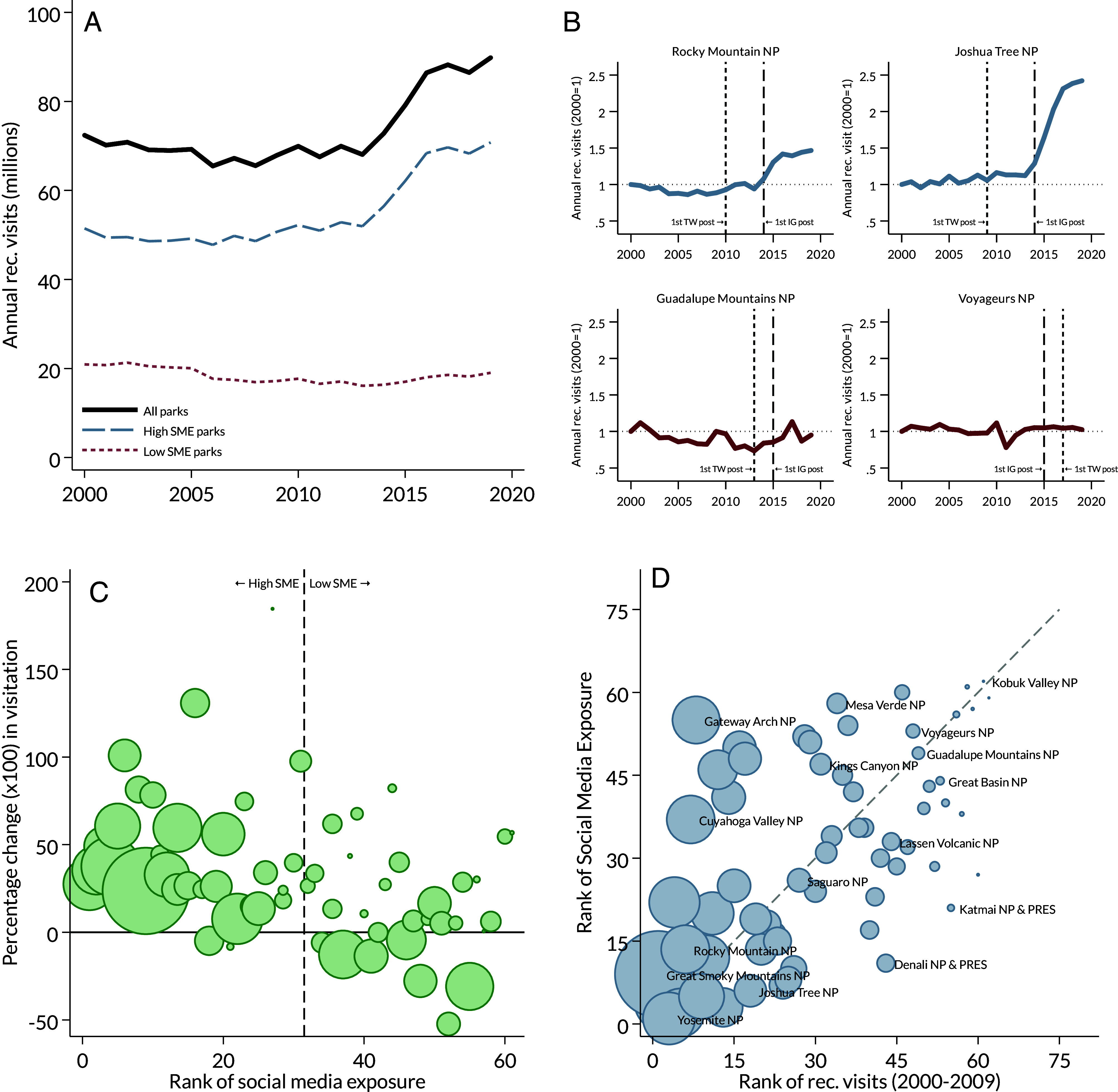
Recreational visits to National Parks and social media exposure (SME) (*A*) Annual recreational visits to all National Parks and disaggregated by parks in *Top/Bottom* half of SME distribution. (*B*) Annual recreational visits for two high SME and two low SME parks (indexed to year 2000). (*C*) Percentage change in annual average visitation before and after social media exposure (from 2005 to 2009 to 2015 to 2019) relative to SME rank. The size of the circle indicates the relative size of mean annual visitation for 2000 to 2009 for each NPS site. Two observations with percentage changes exceeding 200% are excluded from the figure: Kobuk Valley NP, ranked 62nd in SME, had a 400.3% increase in visitation, and National Park of American Samoa, ranked 59th in SME, had a 975.8% increase in visitation. (*D*) Correlation between baseline recreational visitation distribution and social media exposure index. The size of the circle indicates the relative size of mean annual visitation for 2000 to 2009 for each NPS site. The dashed line is the 45-degree line. Labels are shown for select parks.

The US National Park Service (NPS) system was established in 1916 to conserve natural and historic scenery and wildlife for the enjoyment of future generations (1). Since its inception, the NPS system has operated under sometimes conflicting goals of preserving natural landscapes and wildlife habitats while simultaneously making them available for public access and recreation. This tension between preservation and recreation has become more pronounced as visitation to US National Parks has increased dramatically in the last decade. Visitation grew by more than 20 million annual trips in the latter half of the 2010s relative to pre-2010 levels, which averaged around 70 million annual trips (Fig. 1*A*).

Many stories in the popular media have attributed the uptick in National Park visitation to increased social media exposure (2–4). Media has long been influential in the NPS’ history. Experts speculate that Carleton Watkins’ photographs of Yosemite Valley led to Abraham Lincoln’s passing of the Yosemite Grant Act in 1864, which paved the way for preservation of Yellowstone National Park, and the ultimate establishment of the NPS itself (5). Further, the notion that shared media influences visitation to National Parks is not new. In 1951, Time Magazine published Ansel Adams’ photographs of Capitol Reef and Yosemite National Park with the following description: “No artist has pictured the magnificence of the western states more eloquently than photographer Ansel Adams. This summer thousands upon thousands of tourists will follow Adam’s well-beaten trail up and down the National Parks fixing the cold eyes of their cameras on the same splendors he has photographed–and hoping, somehow, to match his art.” (“Art: Realism With Reverence,” Time Magazine, June 4, 1951, Issue 69). Modern advancements in technology have harnessed social networks to give tourists the ability to easily share their visit to a National Park with hundreds of millions of people across the world. Despite the historical importance of media consumption in spurring visitation to National Parks, very little work has been done in quantifying the effect of media exposure on National Park visitation.

Theoretically, the impact of social media is ambiguous. Social media exposure can act as advertising for NPS sites, which can increase visitation. On the other hand, social media may increase virtual (or online) enjoyment of NPS sites, potentially substituting for in-person visits. If social media increases visitation, then this increased demand can generate private and social costs such as overcrowding, congestion, and environmental degradation (6–9). These costs need to be balanced against the enjoyment benefits from increased visitation (10), increases in revenue for the NPS—which may help alleviate roughly $22 billion in deferred maintenance costs for the NPS (11)—and associated increases in local economic activity proximate to park locations (12–15). Despite anecdotal evidence of the link between social media and National Park visitation, there are no credible estimates of the degree to which social media exposure influences visitation at National Parks, nor any evidence on what type of media exposure matters and for what parks.

In this paper, I estimate the relationship between monthly recreational visitation at National Parks and a measure of relative social media exposure. To measure exposure, I gather data from official social media accounts on Twitter and Instagram for each National Park site as well as user-generated media on these platforms. Using these data, I construct a social media exposure (SME) index to test whether parks with greater social media presence incur larger increases in visitation in the 2010s. Each park’s exposure index is based on the rank of the number of Twitter and Instagram followers, the total number of Instagram posts that contain a hashtag with (a variant of) the site’s name, and the total number of “likes” and “retweets” the site’s Twitter account receives. Of course, social media exposure and contemporaneous visitation of a park are likely to be correlated. I account for this endogenous relationship by adopting an instrumental variables strategy that predicts social media exposure using a park’s online popularity—measured using Google search intensity—prior to the introduction of social media. Additionally, park-by-month fixed effects are included in all regressions, which amounts to relying on within-park-month variation in visitation (e.g., comparing visitation to Yosemite in June 2018 relative to the typical June visitation in Yosemite) for estimating the response to SME.

Using this framework, I show that the vast majority of the increase in park-specific visitation is concentrated at parks with the greatest social media exposure. National Parks in the top half of the SME distribution exhibit increases in visitation 16 to 22% greater than parks in the lower half of the distribution. The evidence for changes in low-exposure parks is mixed, suggesting no change or declines in average visitation during the social media era. Additionally, these results suggest large relative increases in annual revenue for parks that see large increases in visitation, which arise predominantly from entrance fees and annual/senior pass sales. In a supplementary analysis that circumvents potential endogeneity concerns with the SME index, I show that time-varying social media exposure—measured by the number of user-generated tweets that mention each park in the preceding 12 mo—influences visitation as well. Tweets that include media attachments (i.e., photographs or videos) are an important predictor of increases in visitation, while negative tweets based on the VADER sentiment lexicon (16) are important predictors of relative decreases in visitation.

Understanding the drivers of recreational visits to National Parks, and public lands more generally, is important for characterizing the costs and benefits of environmental preservation on public lands and policies that attempt to manage or limit visitation (10, 17–20). The degree to which social media, in particular, affects recreation decisions as a driver of demand, and its resultant impact on park revenue, is important as the NPS deals with funding challenges as a result of deferred maintenance, overcrowding, and congestion (8, 11, 19, 21). New sources of social media data are being used to generate more comprehensive estimates of visitation to public lands and to value environmental quality improvements (22–29). Further, recent research shows that geo-referenced Instagram posts led to increases in visitation in the Oregon State Park system for sites that have iconic landscapes (30). This paper builds on this line of research by demonstrating that social media can dramatically influence National Park visitation both positively and negatively, which provides useful insights for managing park-specific operations and understanding how social media shifts demand for recreation.

## Results

### National Park Visitation Increased Dramatically during the 2010s.

Total visitation to the United States’ 62 National Parks increased, unconditionally, by 22% between 2005 and 2009 and 2015 and 2019.^*^Fig. 1*A*, illustrates this trend. Annual recreational visits to all National Parks totaled between 65 and 72 million per year and remained relatively flat, or decreased slightly, until 2013. Between 2013 and 2017, visitation increased quickly to nearly 90 million annual visitors, which persisted through 2019. Table SI2 estimates these changes in a regression framework, which provides corroborative evidence of a 20 to 30% increase in visitation in the post-2015 period even after accounting for unobserved park attributes that vary by month-of-year and weather and economic controls. This increase in visitation could be interpreted as the system-wide impact of social media on visitation, controlling for unobserved park-specific heterogeneity, weather, and local economic conditions, however, this interpretation is complicated by potential confounding variation over time (e.g., due to the NPS Centennial in 2016).

### Increases in Visitation Are Concentrated in Parks with Greater Social Media Exposure.

Fig. 1*A*, shows the trend in visitation for both high and low SME parks. Low SME parks demonstrate a nearly flat trend for the entire sample period, with a slight decrease from 2000 to 2013 and a slight increase from 2013 to 2019. High SME parks demonstrate very little change in visitation until 2013, at which point there is a sharp increase that mirrors the aggregate increase in visitation. These trends suggest that the increase in aggregate visitation is predominantly driven by parks with high social media exposure. These trends are further illustrated in Fig. 1*B*, which shows visitation trends for two high SME parks (Rocky Mountain NP and Joshua Tree NP) and two low SME parks (Guadalupe Mountains NP and Voyageurs NP) relative to each park’s first post on Twitter and Instagram. The high SME parks demonstrate a relatively flat trend until 2013 or 2014, after which visitation increases sharply. For Joshua Tree NP, visitation more than doubles from its 2000 value, whereas Rocky Mountain NP exhibits a 50% increase. Notably, these increases come shortly after each park made their first post on Instagram (and several years after their first post on Twitter). For the low SME parks, there is some year-to-year variation, but very little change in visitation from 2000 levels. These parks were also later adopters of social media.

The correlation between social media exposure and changes in visitation is demonstrated clearly in Fig. 1*C*, where unconditional percentage changes in visitation (i.e., the percentage change in average visitation from 2005 to 2009 relative to 2015 to 2019) are plotted for each park along the distribution of the SME index. Nearly all high SME parks, which generally have greater historical visitation, exhibit increases in visitation. For parks ranked in the lower half of the SME distribution, changes in visitation are more varied, with some relatively large parks showing decreases in visitation. Additionally, Fig. 1*D*, shows the correlation between the SME rank and the rank of baseline visitation, which is positive, but not perfect, suggesting that media exposure does not simply reflect visitation rates.

*SI Appendix*, Fig. S1 shows that the increases in visitation observed in Fig. 1*A*, can be replicated by segmenting aggregate visitation by each of the features that are used to calculate the SME index. Further, *SI Appendix*, Fig. S2*A*, shows that increases in visits are predominantly driven by parks in the top quartile of the SME distribution. The second quartile exhibits smaller increases, while the bottom two quartiles are relatively flat. *SI Appendix*, Fig. S2*B*, shows that there is no corresponding change in nonrecreational visits (e.g., visits from guides, contractors, commuters on NPS roads, outside researchers) across quartiles of social media exposure, which implies that the relative increase in visitation by SME is driven by factors influencing demand for recreational trips.

### Parks with High Social Media Exposure Exhibit 16 to 22% Increases in Visitation Relative to Parks with Low Social Media Exposure, Accounting for Unobserved Park-Specific Heterogeneity and Potential Endogeneity of the Exposure Index.

Panels A and B in Table 1 present estimates from a panel regression of the inverse hyperbolic sine of monthly visitation at a given park regressed on indicator variables for different “post” periods reflecting the social media era interacted with an indicator variable for high SME parks (*Materials and Methods*). All regressions include local weather and economic controls and common year fixed effects. Park-by-month fixed effects are included to absorb seasonal park-specific unobserved heterogeneity, such that identification of changes in visitation arises from within park-by-month comparisons (i.e., comparing visitation to Yosemite NP in June 2017 to the typical June for Yosemite NP within the sample) for high SME parks relative to low SME parks. This estimation strategy accounts for the uniqueness of individual parks that remains fixed over the sample period.

**Table 1.** Effect of social media exposure (SME) on National Park visitation and revenue

	Post 2010	Post 2015	Post Twitter	Post Instagram
*Panel A: OLS estimates for sinh^−1^(visits)*
1[Post]			−0.16*** (0.04)	−0.02 (0.05)
$1\left[\;\text{Post}\right]\times 1\left[\;\text{High}\;{\text{SME}}_{i}\right]$	0.26*** (0.03)	0.28*** (0.03)	0.31*** (0.04)	0.19*** (0.03)
Obs.	14,141	14,141	14,141	14,141
R2 (adj.)	0.94	0.94	0.94	0.94
*Panel B: IV estimates for sinh^−1^(visits)*				
1[Post]			−0.10** (0.05)	0.00 (0.06)
$1\left[\;\text{Post}\right]\times 1\left[\hat{\;\text{High}\;{\text{SME}}_{i}}\right]$	0.17*** (0.05)	0.15** (0.06)	0.20*** (0.06)	0.16*** (0.05)
Obs.	14,141	14,141	14,141	14,141
R2 (centered)	0.02	0.02	0.02	0.01
Kleibergen–Paap F statistic	373.1	382.9	399.2	522.9
*Panel C: OLS estimates for annual revenue*				
1[Post]			−0.64** (0.23)	−1.10** (0.44)
$1\left[\;\text{Post}\right]\times 1\left[\;\text{High}\;{\text{SME}}_{i}\right]$	0.87** (0.36)	1.77*** (0.40)	1.08*** (0.35)	1.69*** (0.36)
Obs.	791	791	791	791
R2 (adj.)	0.97	0.97	0.97	0.97
*Panel D: IV estimates for annual revenue*				
1[Post]			−1.06*** (0.35)	−1.78** (0.65)
$1\left[\;\text{Post}\right]\times 1\left[\hat{\;\text{High}\;{\text{SME}}_{i}}\right]$	1.41** (0.59)	3.16*** (0.78)	1.84*** (0.59)	2.76*** (0.65)
Obs.	791	791	791	791
R2 (centered)	0.07	0.12	0.08	0.14
Kleibergen–Paap F statistic	29.7	26.4	29.9	40.5

Notes: Panels A and B: The dependent variable is the inverse-hyperbolic sine of monthly recreation visits at National Park sites between 2000 and 2019. Panels C and D: The dependent variable is the annual total revenue (in millions of 2019$) at National Park sites between 2004 and 2019. Each column reflects a different definition of 1[Post]: “Post 2010” equals one for all time periods ≥ 2010; “Post 2015” equals one for all time periods ≥ 2015; “Post Twitter” equals one for all time periods after the park’s first tweet; “Post Instagram” equals one for all time periods after the park created its Instagram account. All specifications include county-level temperature and precipitation, state-level real per capita income, and state-level unemployment rate. Panels A and B include park-by-month fixed effects and year fixed effects. SEs are two-way clustered at the park-by-month and year-by-month level. Panels C and D include park fixed effects and year fixed effects. SEs are two-way clustered at the park and year level. ^∗^, ^∗∗^, and ^∗∗∗^ represents significance at the P<0.1, P<0.05, and P<0.01 level.

Panel A of Table 1 presents ordinary least squares (OLS) estimates for high SME parks interacted with four periods reflecting the social media era. Estimated coefficients are 0.19 to 0.31 for high SME parks, indicating that high SME parks see 21 to 36% increases in visitation relative to low SME parks.^†^ For periods after each park’s first Twitter post, low SME parks exhibit decreases in visitation, while for periods after each park’s first Instagram post, low SME parks exhibit no statistical change in visitation. Twitter posts generally occur earlier in the sample (around 2010), which aligns with a relative decline in visitation in the early 2010s for low SME parks (Fig. 1*A*), whereas Instagram posts generally occur later in the sample (around 2015) (*SI Appendix*, Fig. S3). These coefficients are absorbed by year fixed effects in the specifications with park-invariant post periods.

The coefficient estimates in panel A provide evidence that increases in visitation is concentrated at parks with greater social media exposure. This descriptive result is interesting in its own right; however, these results may be endogenous for two reasons. First, SME is measured using static features at the end of the sample and thus may be driven by reverse causality in which visits in earlier time periods generate more social media exposure. Second, SME may be correlated with unobserved, time-varying park-specific attributes (e.g., if more heavily visited parks hire a more effective social media manager to manage the park’s online presence). To deal with this potential endogeneity, I define an instrumental variable (IV) based on relative Google search intensity for each park in the 2004 to 2009 period, which I use to predict which parks will have high social media exposure in later years (*Materials and Methods*). Google search intensity in 2004 to 2009 is correlated with SME but does not directly influence park visitation in the post period, conditional on year and park-by-month fixed effects as well as other controls (*SI Appendix*, Fig. S4). The instrument would be invalid if pre-treatment online search intensity was correlated with other unobserved, time-varying variables that generated more visitation in the post-treatment period, which is evaluated in more detail in the *Discussion*. The IV strategy accounts for the first problem of reverse causality. The second problem of unobserved, time-varying park-specific attributes is addressed by including year fixed effects in all specifications and exploring the sensitivity to known time-variant shocks in subsequent analyses.

Table 1, panel B, presents IV estimates. The IV coefficients are smaller than the OLS coefficients, suggesting that the correlation between SME and the residual error term in Panel A is positive, leading those estimates to be biased away from zero. The smaller IV estimates range from 0.15 to 0.20, all of which are statistically different from zero at the P<0.05 level, suggesting a 16 to 22% increase in visitation for high SME parks relative to low SME parks. The instrument is strong, based on first-stage F-statistics exceeding 300 in all specifications. Despite the smaller point estimates from the IV strategy, the 95% CIs for all coefficients on the interaction between the post period and high SME in panels A and B overlap. The coefficient on the baseline post-Twitter period is negative and statistically significant at the P<0.05 level (column 3), suggesting that low SME parks see average decreases in visitation after each park’s first tweet. The same coefficient for low SME parks after their first Instagram post is statistically indistinguishable from zero (column 4).

*SI Appendix*, Fig. S5 decomposes these average coefficients within an annual event study framework relative to the year 2009 for both high and low SME parks (panel A), as well as the difference between the two groups (panel B). This framework illustrates how changes in visitation as a result of media exposure evolve over time. In years 2005 to 2008, changes in visitation rates are similar for low and high SME parks and statistically similar to zero (except for low SME parks in 2005, which is statistically positive but not statistically different from the estimate for high SME parks). Between 2010 and 2013, during the early years of the social media era, high SME parks’ visitation rates remain similar to that of 2009, while low SME parks’ visitation rates vary from no change to relative decreases. The gap between low and high SME parks begins to widen in 2014 and persists through the end of the sample. Removing two low SME parks with extreme percentage changes in visitation (panels C and D) leads to similar conclusions, with a more stable increase in visitation beginning in 2013. Jointly, these coefficients suggest that a) high and low SME parks had similar trends prior to the social media era; b) low SME parks saw decreases in visitation in the early 2010s, whereas high SME parks’ visitation remained unchanged; and c) low SME parks exhibit increases in visitation in later years of the sample, but the increases in visitation for high SME parks were proportionally larger, leading to larger increases for high SME parks.

### Social Media Exposure Leads to Large Increases in Revenue for Individual Parks.

The increases in visitation at high SME parks can lead to increases in revenue directly through entrance fees, annual pass purchases, and recreation and camping fees. In Table 1, panels C and D, I estimate changes in revenue directly by park-specific annual revenue on indicators for the post period interacted with an indicator for high SME parks (*Materials and Methods*). The OLS coefficients in panel C suggest that the average high SME park yields an additional $0.9M to $1.8M in annual park revenue relative to the typical low SME park. IV estimates in panel D suggest even larger changes in revenue for high SME parks, ranging from $1.4M to $3.2M in additional revenue per year relative to the typical low SME park. These estimates reflect not only which parks charge entrance fees (high SME parks are more likely to have entrance fees), but also the prevailing differences in aggregate visitation (i.e., changes in levels as opposed to percentage changes). *SI Appendix*, Fig. S6 shows that the increase in revenue for high SME parks is dominated by entrance fee revenue as well as pass sales. Recreation and camping fees trend similarly between high SME and low SME parks and decline in later years of the sample. Because both baseline visitation and the change in visitation at high SME parks is large, the commensurate change in revenue is large. Using the Post-2015 coefficient, the average high SME park raised an additional $15.8M (=$3.16M per year × 5 y) during the post-treatment period relative to low SME parks.

### The Relative Increase in Visitation for Parks with High Social Media Exposure Is Heterogeneous across Region and Park Characteristics.

**Fig. 2.**
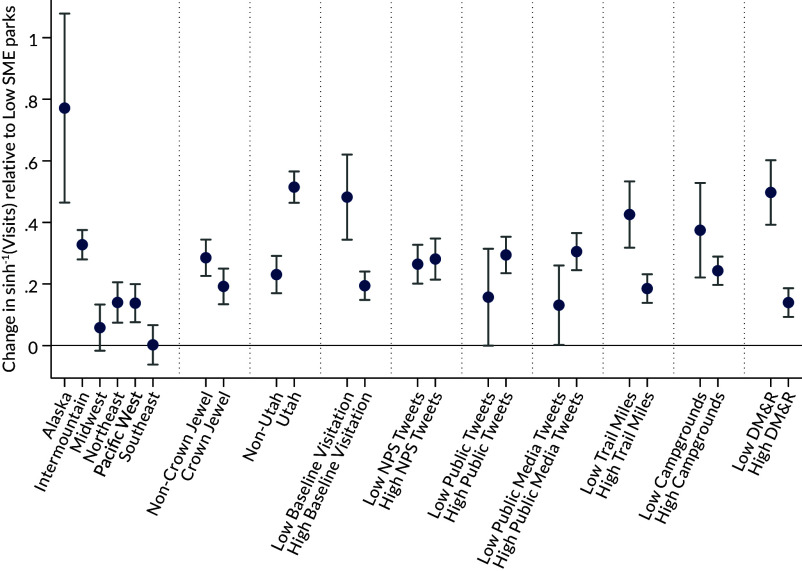
Heterogeneity in SME effect on visitation by park characteristics. Each set of coefficients represents estimates from ten different regression specifications where the heterogeneous effects are identified via interactions with $1\left[\;\text{Post}{\text{2015}}_{t}\right]\times 1\left[\;\text{High}\;{\text{SME}}_{i}\right]$. The figure shows results for Post 2015. Coefficients represent changes in monthly visitation (transformed via the inverse hyperbolic sine) for high SME parks relative to the average low SME park. 90% CIs are shown in brackets. All regressions include park-by-month and year fixed effects and weather and economic controls. The first set of results displays regional heterogeneity. Crown Jewel equals 1 for Yosemite NP, Yellowstone NP, and Grand Canyon NP. Utah equals 1 for any park in Utah. High/low baseline visitation is whether park is above/below median visitation in 2000 to 2009. High/low NPS tweets is whether park is above/below median number of tweets from official NPS account in 2000 to 2019. High/low public tweets is whether park is above/below median number of tweets from non-NPS accounts in 2000 to 2019. High/low Public Media Tweets is whether park is above/below median number of tweets from non-NPS accounts with media attached in 2000 to 2019. High/low trail miles is whether park is above/below median number of hiking trail miles within park. High/low campgrounds is whether park is above/below median number of campgrounds within park. High/low deferred maintenance is whether park is above/below median deferred maintenance in FY2022.

Fig. 2 shows the relative change in visitation among high SME parks relative to low SME parks, broken out by park characteristics for the post-2015 period. High SME parks in Alaska and the Intermountain region exhibit the largest increases, whereas changes in visitation for high SME parks in the Midwest and Southeast are statistically similar to that of low SME parks. For the three “Crown Jewel” parks (i.e., Yosemite NP, Yellowstone NP, and Grand Canyon NP), the relative increase in visitation is smaller, although statistically similar to, the typical non-Crown Jewel high SME parks. Parks in Utah, all of which are high SME, have much larger increases in visitation (coefficient: 0.51) relative to low SME parks (coefficient: 0.23) than the typical non-Utah park. Utah parks are broken out because Utah has the highest density of parks with high SME in a small geographic area and implemented a statewide “Mighty Five” media campaign to increase tourism in the mid-2010s.

Fig. 2 also shows heterogeneity by park characteristics which are summarized by whether the park is above/below the median park for the given characteristic. Parks with high baseline visitation (2000 to 2009) exhibit smaller increases in visitation relative to parks with low baseline visitation, which suggests SME exposure may increase visibility for previously less-visited parks. The quantity of tweets coming from the park’s official Twitter account is inconsequential for relative increases in visitation. In contrast, the quantity of public tweets and public tweets with media attachments lead to relatively larger increases in visitation for high SME parks relative to low SME parks. These results suggest that the quantity of tweets about a park, rather than the quantity of tweets from the park’s official account, is a better predictor of increases in visitation. High SME parks with more miles of trails and more campgrounds see fewer relative visits compared to low SME parks, indicating that more extensive recreational opportunities may not complement social media exposure. Last, high SME parks with higher deferred maintenance and repairs (DM&R) costs see smaller relative increases in visitation than high SME parks with low DM&R costs, which suggests that parks that need increased revenue to address DM&R may not benefit as much from social media exposure. All of these results are similar when using the “post-Instagram” period as the treatment period (*SI Appendix*, Fig. S7).

### Increases in Park-Specific Social Media Posts in the Previous Year Lead to Increases in Visitation. Posts with Attached Media Amplify Visitation Rates, While Posts with Negative Sentiment Attenuate Visitation Rates.

The finding that increases in visitation are concentrated at high SME parks is based on a discretized, static measure of exposure. Table 2 shows that visitation responds to a complementary time-varying measure of media exposure, as well, and explores various characteristics associated with this alternative measure of exposure. I construct standardized measures of the number of tweets that include the park’s username in the text of the tweet in the preceding 12 mo, in addition to measurable attributes of those tweets (*Materials and Methods*). Column (1) shows that a one-standard-deviation increase in the quantity of user tweets translates to an approximately 2% increase in monthly park visits. In columns (2) and (3), I interact the baseline quantity of tweets with the aggregate quantity of retweets and likes those tweets generated. Neither of the coefficients on the interaction terms for either retweets or likes are significant, suggesting that the quantity of tweets matters more than measures of social media engagement. Column (4) shows that tweets with attached media increase visitation by approximately 4% for every SD increase. The baseline coefficient on the aggregate quantity of tweets is small, negative, and insignificant. This result suggests that the quantity of images and videos posted about the park substantially increases visitation relative to tweets without media attachments. In column (6), tweets with positive or neutral sentiment, as measured by the VADER sentiment lexicon (16), lead to 9% increases in visitation for every SD increase. Tweets with negative sentiment moderate this increase: Every SD increase in negative tweets leads to a relative 6% decrease in visitation. In *SI Appendix*, Table S3, I provide examples of tweets that are associated with increases in visitation (e.g., tweets with positive/neutral/negative sentiment). Overall, these results suggest that using the quantity of tweets about a park in the previous 12 mo as a measure of exposure leads to increases in visitation, but the content associated with the exposure (e.g., images of the park) and the sentiment of the exposure (i.e., positive or negative) are critical for understanding whether, and in what direction, social media can affect demand for National Park visitation.

**Table 2.** Changes in visitation in response to quantity and characteristics of user tweets

	(1)	(2)	(3)	(4)	(5)
No. original user tweets in prev. 12 mo (std)	0.02*** (0.01)	0.04*** (0.01)	0.02 (0.01)	−0.01 (0.01)	0.09*** (0.01)
No. retweets in prev. 12 mo (std)		−0.01 (0.01)			
No. of likes in prev. 12 mo (std)			0.00 (0.01)		
No. of user tweets with media in prev. 12 mo (std)				0.04*** (0.01)	
No. of negative user tweets in prev. 12 mo (std)					−0.06*** (0.01)
Obs.	14,141	14,141	14,141	14,141	14,141
R2	0.94	0.94	0.94	0.94	0.94
Park-by-month FEs?	Y	Y	Y	Y	Y
Year FEs?	Y	Y	Y	Y	Y
Controls?	Y	Y	Y	Y	Y

Notes: The dependent variable is the inverse-hyperbolic sine of monthly recreation visits at National Park sites. Original user tweets are the universe of tweets that contain the NPS site’s Twitter handle (e.g., “@YosemiteNPS”) and summed over the preceding 12 mo. Retweets, likes, and user tweets with media indicate the counts of original user tweets that are retweeted, liked, and contain attached media (e.g., photos). Negative user tweets are the total number of tweets with a negative VADER sentiment score. Independent variables presented are standardized (std) to be mean zero with unit changes reflecting one SD. Controls include county-level temperature and precipitation, state-level real per capita income, and state-level unemployment rate. SEs are two-way clustered at the park-by-month and year-by-month level. ^∗^, ^∗∗^, and ^∗∗∗^ represents significance at the P<0.1, P<0.05, and P<0.01 level.

This dynamic analysis complements the results based on the static SME index and sheds additional light on the mechanisms through which social media exposure influences visitation. The results in Table 2 suggest that the quantity of social media posts about park is a good summary measure of exposure, which are related to the components of the SME index (i.e., number of Instagram hashtags or number of Twitter “retweets”). As shown in Fig. 2, the number of public tweets and number of public tweets with media attachments are influential for changes in visitation among high SME parks. All but four high SME parks are in the top half of the high public tweets distribution and all but five high SME parks are in the top half of the high public tweets with media attachments distribution, which suggests that the measures of exposure are in strong agreement.

### The Relative Increase in Visitation for National Parks with High Social Media Exposure Is Insensitive to Sample Restrictions, Removing the Year of the National Park Service Centennial, and Alternative Specifications.

**Fig. 3.**
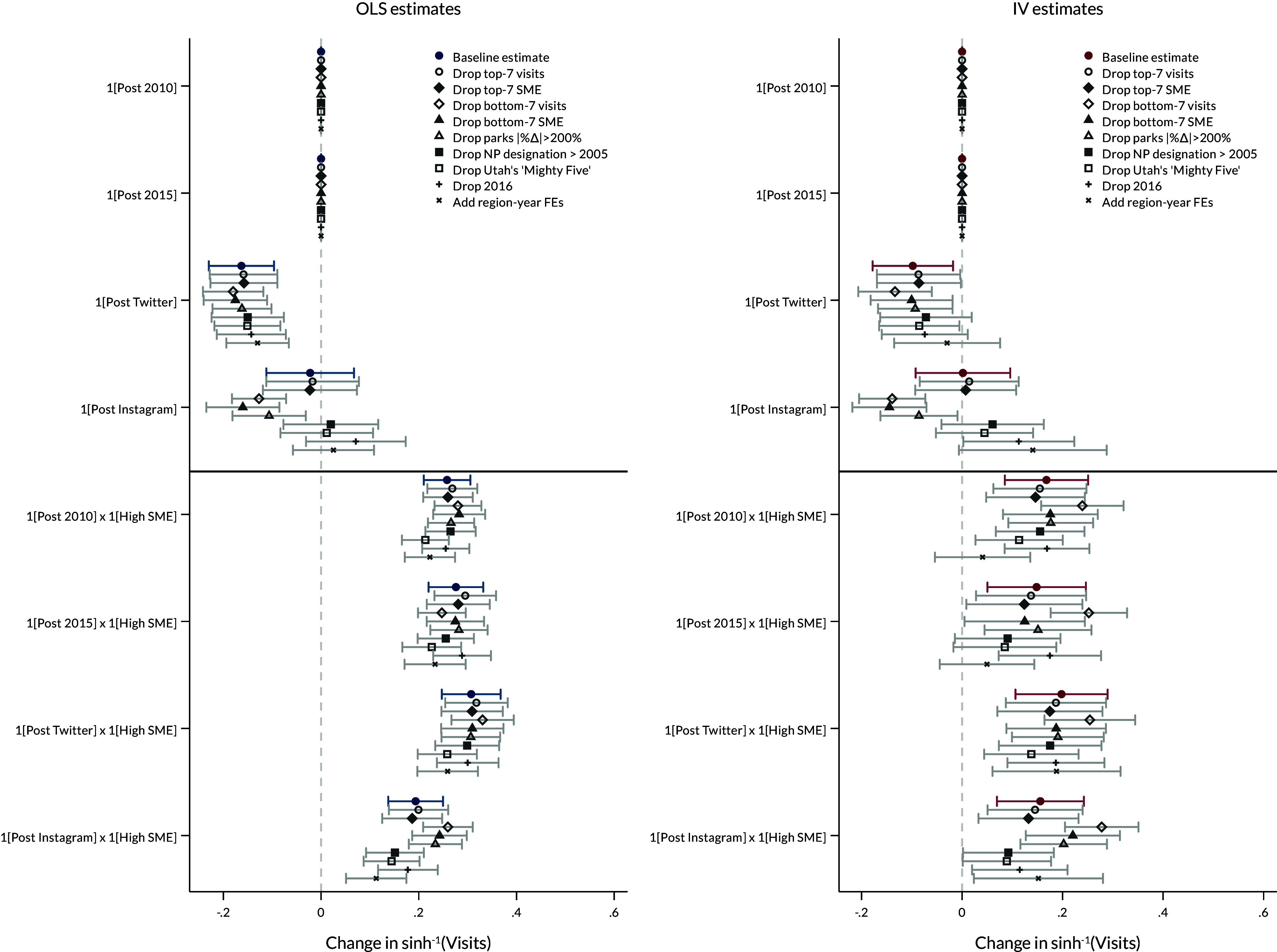
Specification chart of primary estimates for various sample restrictions and robustness checks. Each set of coefficients represents estimates from nine different regression specifications. The dependent variable is the inverse hyperbolic sine of monthly visitation at each National Park site in each specification. “Drop top-7 visits” removes parks ranked in the top 7 of visitation during the 2000 to 2009 period. “Drop top-7 SME” removes parks ranked in the top 7 of social media exposure. The “bottom-7” specifications are analogous, but remove the bottom 7 parks. “Drop 2016” removes the year of 2016 from the regression to account for the NPS Centennial. “Drop parks |%Δ|>0.75” drops three low SME parks that had greater than 75% changes in visitation from 2005 to 2009 to 2015 to 2019. “Drop NP designation > 2005” drops four parks that received designation as National Parks after 2005. “Drop Utah’s ‘Mighty Five”’ drops all five parks in Utah that had a contemporaneous state media campaign. “Add region-year FEs” substitutes region-by-year fixed effects for year fixed effects in the regression. Note that the base coefficient is unidentified for 1[Post 2010] and 1[Post 2015] because of perfect collinearity with year fixed effects. Whiskers represent 90% CIs; SEs are two-way clustered at the park-by-month and year-by-month level.

The relative increase in visitation for high SME parks might be driven by parks in the extremes of the visitation or the exposure distribution. One concern might be that the NPS “crown jewel” parks generate a lot of visitation and, thus, social media may simply amplify the visibility of these popular parks. Additionally, some low SME parks exhibit dramatic changes in visitation (Fig. 1*C*), which could have a large influence on average effects. Further, 2016 was the NPS Centennial, during which the National Park Foundation spearheaded the Find Your Park campaign to spur visitation. Although the primary regressions control for common year fixed effects and the Centennial was likely intended to stimulate visitation at all parks (not just high SME parks), the Find Your Park initiative was largely conducted as a social media campaign that may have had differential effects across parks.^‡^Fig. 3 shows that the primary OLS and IV estimates are generally insensitive to a) removing the top-seven parks in terms of baseline visitation and the SME index; b) removing the bottom-seven parks in both baseline visitation and the SME index; c) removing parks with absolute changes in visitation rates exceeding 75%; d) removing three parks that received their National Park designation in 2005 or later; e) removing the “Mighty Five” Utah parks that had a contemporaneous statewide advertising campaign, and f) removing 2016—the year of NPS Centennial—from the analysis. The coefficient capturing the relative change in visitation for high SME parks is insensitive to these sample restrictions for both OLS and IV specifications. The baseline coefficient for the post-Instagram variable captures the average effects for low SME parks after their first social media post. This effect is significantly negative when the bottom seven parks in terms of SME or historical visitation are removed from the sample, or when removing three parks with extreme percentage changes in visitation. Further, specifications that include region-by-year fixed effects, rather than year fixed effects, result in smaller and insignificant changes for the high SME parks in the IV specifications for both post-2010 and post-2015 periods, but not for the post-Twitter and post-Instagram specifications. This result suggests that some variation in the park-invariant post periods may be explained by regional, annual shocks.

## Discussion

Using a dataset that matches social media exposure with National Park visitation, I show that parks with greater exposure saw dramatic increases in recreational visitation over the last decade. On average, parks with greater exposure exhibit 16 to 22% increases in recreational visits, whereas parks with weaker exposure exhibit no change, or decreases, in visitation. The trends between low and high SME parks begin to diverge in the early-to-mid-2010s, which is when social media platforms gained prominence, with a wedge that persists through the end of the sample.

One concern might be that popular parks simply have popular social media presences, leading to higher visitation and higher exposure. Fig. 1*D*, suggests that this is not necessarily the case. The correlation between visitation and social media exposure is positive, but not perfect. Additionally, all regression specifications contain park-by-month fixed effects, which control for unobserved attributes of each park that vary by month. So, unobserved, time-invariant qualities of the park that drive visitation (including, e.g., baseline popularity or the unique landscape of the Grand Canyon) are differenced out of the regression, allowing for within-park changes in visitation before/after the social media era to be compared across parks with differential social media exposure. Moreover, Fig. 2 shows that more heavily visited high SME parks in 2000 to 2009 have relatively smaller increases in visitation than parks with lower baseline visitation rates. The same story is true for the “crown jewel” parks. Additionally, as shown in Fig. 3, the primary results are robust to removing the top parks in terms of visitation and social media exposure, which alleviates concerns that the results are driven solely by parks that are highly popular or visible at baseline.

Additionally, the SME index is defined using ex post characteristics of an individual park’s social media presence, which may present complications for interpreting the differences between high and low SME parks as causal. The IV strategy mitigates these concerns by leveraging a measure of pre-social media online popularity to predict social media exposure in later years. Park-by-month fixed effects are included in these IV specifications, so using online popularity to predict exposure is also conditioned on unobserved, time-invariant park attributes. One pathway for the validity of the instrument to fail is if the instrument is correlated with some other unobserved, dynamic component of visitation. One potential threat is a “contagion” effect whereby parks that are popular online (indicated by 2004 to 2009 Google search intensity) generate additional visits via network effects leading them to become more popular over time. A dynamic effect of this sort might weaken the credibility of the Google search intensity instrument, although there is no evidence that dynamic increases in visitation of this sort were occurring prior to the uptick around 2013 for parks with high Google search intensity (*SI Appendix*, Fig. S8).

Beyond the descriptive evidence that increases in visitation are concentrated among parks with high social media exposure and the IV strategy that leverages online popularity in the pre-social media era to estimate differential effects of social media exposure, I provide additional evidence that media exposure and visitation to National Parks is linked using a different dataset that circumvents the potentially endogenous SME index. The results in Table 2 buttress the primary findings: Parks that have more user-generated tweets in the preceding year see increases in visitation. This additional analysis permits examination of which social media characteristics drive changes in park-specific visitation. Measures of engagement on social media (likes and retweets) are not influential, but the quantity of media attachments and the sentiment of social media posts matter for visitation. These results are sensible. Twitter was primarily designed as a micro-blogging website for sharing text, whereas Instagram and other social media platforms are designed for sharing images and/or videos. The attached media content on Twitter likely serves as a good proxy for these other platforms as well. Additionally, not all media exposure is good exposure. Tweets with negative sentiment reduce visitation, which is sensible and reassuring for the empirical design. For example, social media posts about traffic congestion in parks, low visibility due to poor air quality (17), or forest fire risk (32) may discourage visitation.

Increases in visitation can generate large increases in revenue for individual parks and the NPS as a whole. According to the Federal Lands Recreation Enhancement Act (33), the NPS site can retain 80% of the revenue generated from entrance fees and other recreational fees, with the remainder redistributed within the agency. However, these visitation increases also generate additional costs for each site (e.g., adding additional stress to facilities and physical infrastructure, eroding hiking trails, generating traffic congestion). A natural question is whether the increases in visitation are targeted to parks with greater levels of deferred maintenance costs. *SI Appendix*, Fig. S9 shows that the SME index and the distribution of deferred maintenance and repairs per visitor are very weakly correlated and Fig. 2 shows that high SME parks with high deferred maintenance costs see relatively smaller increases in visitation. Both results suggest that social media has poorly targeted revenue increases to parks that need it most.

This paper provides evidence that greater social media exposure leads to relative increases in National Park visitation. A natural question is what mechanism(s) might be driving this effect. Although beyond the scope of this paper, a recreation demand model might incorporate social media as form of advertising, which would lower the implicit price of a trip to a given park. This advertising could be informative (i.e., the NPS alerting potential visitors about road closures) or persuasive (i.e., an Instagram influencer posting a selfie at sunset among the Joshua Tree NP landscape) and those channels might have different effects on visitation. Moreover, this social-media-as-advertising might be a function of visitation itself. That is, part of the enjoyment of a trip to a National Park might be sharing that trip with others on social media, which begets additional visits from those in the sharer’s network. Unpacking these mechanisms, and the embedded network effects, is a promising line of future research.

As some parks struggle to manage the increases in visitation, and its associated costs, other parks have seen declines in visitation (perhaps as people substitute toward parks with greater SME). A promising line of future work would consider whether these increases in visitation are, on net, welfare-improving. By generating new park visitors, National Parks can inspire a broader set of the population to appreciate and value environmental preservation. On the other hand, increases in visitation may diminish the quality of trips for some parkgoers and lead to congestion that limits the ability of others to visit. Understanding the social costs and benefits of increases in recreational visits on public lands and the implications of policies intended to manage visitation to some of the world’s most iconic public goods are worthy areas of future inquiry.

## Materials and Methods

### Data.

#### NPS visitation data.

Monthly National Park visitation data were obtained from the NPS visitor use statistics database (https://irma.nps.gov/STATS/) for 1980 to 2019. These data contain monthly counts of recreational visitors, nonrecreational visitors, among other park characteristics. NPS sites are disaggregated into 17 park types, but I focus only on the 62 National Parks (see *SI Appendix*, Table S1 for a list of parks analyzed). I exclude National Recreation Areas, National Battlefields, National Rivers, and so forth. These alternative NPS sites are unlikely to exhibit a strong social media presence and are likely poor comparisons to the more iconic National Parks.

Each park has a fixed public use counting and reporting instruction file available online that describes the park-specific process for measuring recreational visitors. Most parks use inductive loop traffic counter at primary entrances, net out fixed percentages of “non-reportable” vehicles, and scale the number of vehicles by a person-per-vehicle scalar (often between 2 and 3 people per vehicle), which can vary by season or park entrance. Other, often smaller parks, use manual counts of visitors that enter ranger stations or visitor centers and then use a fixed percentage to approximate the number of visitors that did not enter a ranger station or visitor center. There is likely measurement error in the number of visitors; however, the measurement and reporting procedures are largely consistent over time and updated very infrequently. Much of the measurement error is likely absorbed by the park-by-month fixed effects in the regressions.

#### Social media data.

For each National Park, social media accounts were identified manually using a search for the NPS site name on Twitter and Instagram. Linking NPS sites with social media accounts was conducted in June 2020. 3 of the 62 National Parks did not have an official Instagram account (Crater Lake NP, Gates of the Arctic NP & PRES, and Great Basin NP) and 8 of 62 did not have an official Twitter account (Great Sand Dunes NP & PRES, Hot Springs NP, Isle Royale NP, Kings Canyon NP, Kobuk Valley NP, Mesa Verde NP, Virgin Islands NP, and White Sands NP). Sequoia National Park and Kings Canyon National Park have distinct visitation records, although their social media accounts are linked (i.e., @SequoiaKingsNPS). So, some of the social media data linked to a park’s username (e.g., followers on Twitter or Instagram) for these two parks are identical.

For each park with a Twitter account, account information was scraped from the public Twitter API, including the total number of Twitter posts, the total number of Twitter followers, and the date the account was established on Twitter. All of these values are fixed at their June 2020 levels. I also obtained the entire series of Twitter posts—or, tweets—for each NPS site with a Twitter account.^§^ These data contain the timestamp of each post, the post text (including a link to any associated media), whether the post was a reply to another account’s tweet, whether the post was a retweet of another account’s tweet, the number of favorites the tweet generated, the number of retweets the tweet generated. All values are tied to their June 2020 levels.

Additionally, for each NPS site, I manually scraped the number of Instagram posts, the number of Instagram followers, the number of users that the NPS account is following, and the date of the account’s first post. The follower and following counts are current as of June 2020. I also obtain counts of posts that contain a “hashtag” for individual National Parks on Instagram. Hashtags are words or phrases, preceded by “#,” that serve as easy ways to identify messages on a specific topic. For each National Park, I searched for public posts containing a hashtag for variants of the park’s name and obtained the count of posts that matched that search criteria. For example, for Yosemite National Park, I conducted searches for three variants of its name: “#yosemite,” “#yosemitenationalpark,” and “#yosemitenps.” For each park, I aggregate those counts to produce the total number of public posts containing any of those hashtags on Instagram. These counts are totals as of June 2020. Obtaining information on Instagram posts at scale was infeasible due to limited API access; the Instagram data for each park site was hand-collected.

#### Weather data.

I spatially match each park site’s centroid to its county. Park centroids are calculated from shapefiles of park boundaries publicly available online (https://public-nps.opendata.arcgis.com/datasets/nps-boundary-1/explore). I then match monthly temperature and precipitation data from the PRISM dataset (34). From the gridded PRISM data, I find average maximum and minimum temperatures and precipitation for all grid cells that fall within a county’s boundaries. Then, I construct temperature bins for the number of days that the temperature falls within a 10-°C range each month. I aggregate the temperature bins and total precipitation to the county-month level. For noncontiguous states and territories not covered in the PRISM dataset provided by (34) (i.e., American Samoa, Alaska, Hawaii, and the Virgin Islands), I use NOAA’s Global Historical Climate Network-Daily (GHCN-Daily) data. For each park, I find the centroid of the park boundaries and all GHCN-Daily stations within a 50-mile radius. I aggregate the maximum and minimum temperatures and precipitation levels similarly to the PRISM data to construct maximum temperature bins and total precipitation levels at the county-month level.

#### Local economic data and NPS revenue data.

For each state, I assemble economic data as controls for local economic shocks that could influence visitation. I gather nominal annual per capita household income data at the state level from Federal Reserve Economic Data and convert to real 2019 dollars. I also include monthly, seasonally adjusted unemployment rates from the US Bureau of Labor Statistics’ Local Area Unemployment Statistics (https://www.bls.gov/lau/rdscnp16.htm). For the Virgin Islands, I gather unemployment rates from VIeWS (V.I. Electronic Workforce System, https://www.vidolviews.org/vosnet/).

I obtained annual park-specific revenue data from NPS directly. These data include revenue from entrance fees, annual and senior pass sales (both park-specific passes as well as the “America the Beautiful” pass), recreation fees (e.g., backcountry permits), camping fees, and miscellaneous other fees. I convert nominal revenue to real 2019 dollars. I also gather deferred maintenance data for FY2022 for each park from official NPS records.

### Construction of the Social Media Exposure (SME) Index.

Using the social media data, I construct a metric of social media exposure based on the relative rank of several features. First, I rank each park by the following features: 1) number of Twitter followers, 2) number of Instagram followers, 3) total number of Instagram posts with a hashtag corresponding to the site’s name (e.g., “#yellowstone” or “#yellowstoneNPS”), 4) total number of likes (or favorites) a park’s Twitter posts received, and 5) total number of retweets a park’s Twitter posts received. All values are current as of June 2020. These measures are all highly correlated with one another as shown in *SI Appendix*, Fig. S10. Each of these features were chosen as “incoming” measures of social media exposure generated from external sources. That is, a site could have a very active Instagram account that receives little attention, which would not influence the likelihood that changes in social media exposure affect visitation in a meaningful way. In contrast, if a site’s social media presence were large, such that a site’s social media accounts had many followers and substantial engagement on Twitter and Instagram, it is more likely that activity would influence visitation to a specific site. Using these five ranked measures of exposure, I calculate each site’s mean rank, weighting each feature equally, and rank each site according to their mean rank. This process results in the variable, $SME_{i}$, which is defined as a cross-sectional measure of relative social media exposure for each park, i. I further segment this exposure metric into parks with $\;\text{High}\;{\text{SME}}_{i}$ based on whether park i is in the top half of the $SME_{i}$ distribution, indicating relatively stronger social media exposure.

The $SME_{i}$ index is a useful snapshot of the level of media exposure for each park, but it does not vary over time. To construct a time-varying measure of park exposure, I generate a secondary dataset based on user tweets (i.e., tweets from the general public) that include the NPS sites’ Twitter handle—“YosemiteNPS” or “GreatSmokyNPS”—within the content of the tweet. This process results in a dataset of 998,882 tweets, from April 2008 to December 2019. I aggregate these data to the park-by-month level, generating a series of j variables that capture the number of original tweets, the number of retweets, the number of likes a tweet receives, and the number of tweets that had a media attachment (i.e., a photo or video). Additionally, I process the sentiment of the tweet using the VADER lexicon, which provides a sentiment score for each tweet’s text, marking the tweet as positive, neutral, or negative (16). Sentiment analysis has been used in a variety of contexts to measure the effect of temperature on temperament (35), air pollution on well-being (36), and to understand the drivers of donations to public goods (37), among many other applications. See *SI Appendix*, Table S3 for examples of tweets and sentiment scores. I aggregate the number of “positive” tweets (with a VADER score greater than 0) and the number of “negative” tweets (with a VADER score less than 0). From this set of park (i)-by-month-of-sample (t) tweets for each characteristic j (defined above), $Tweets_{\mathrm{it}}^{\;j}$, I sum all tweets in the previous 12 mo to define $Tweets\_12m_{\mathrm{it}}^{j}=\sum_{k=1}^{12}Tweets_{it-k}^{\;j}$. To facilitate interpretation, I standardize this variable to be mean zero with a unit change reflecting one SD.

### Empirical Framework.

To estimate the effect of social media on visitation, I construct several variables that capture the “social-media era.” The first two—$1[Post2010_{t}]$ and $1[Post2015_{t}]$—are crude measures of when Twitter and Instagram, respectively, gained prominence. Twitter was launched in 2006 but did not have a substantial user base until early 2010.^¶^ Further, images were not embedded in tweets until 2010. As shown in *SI Appendix*, Fig. S3, most NPS sites posted their first tweet around 2010, although many sites did not post their first tweet until later in the 2010s. Despite this early adoption, the vast majority of outgoing tweets from NPS Twitter accounts occurred in the mid-2010s. Instagram’s popularity occurred after Twitter. The first Instagram post occurred in summer 2010, although the website became much more popular in the mid-2010s.^#^*SI Appendix*, Fig. S3 shows that the majority of NPS sites began their Instagram accounts in 2015 or later. I use this information to define park-specific indicators—$1[PostTW_{\mathrm{it}}]$ and $1[PostIG_{\mathrm{it}}]$—that define the period after each NPS made its first post on each social media site. These latter two indicators capture the park’s willingness to engage on social media, indicating some expected benefit. Collectively, these four indicators capture the relevant time periods when social media might affect visitation.

To estimate the effect of social media exposure on visitation, I estimate the following equation:

[1] $$\begin{array}{c} sinh^{-1}(V_{ismt})=\beta_{1}1[Post_{(i)t}]+\beta_{2}1[Post_{(i)t}]\times 1[\;\text{High}\;{\text{SME}}_{i}]\\ +\;{\text{controls}}_{ist}+\alpha_{im}+\lambda_{y}+\varepsilon_{ismt}, \end{array}$$

where $V_{\mathrm{ismt}}$ is the number of visits to site i in state s during month-of-year m in month-of-sample t, which is transformed via the inverse hyperbolic sine ($sinh^{-1}$). The inverse hyperbolic sine is used, similar to a logarithm, to facilitate interpretation of coefficients as semielasticities, to reduce the effect of extreme outliers, and because there are sites with zero visits in certain months (31). Because the inverse-hyperbolic sine can be sensitive to scale (38), this equation is replicated with logged outcomes, which drops months with zero visitation, as well as fixed-effects Poisson regressions as robustness checks in *SI Appendix*, Table S4. $1[Post_{(i)t}]$ is a variable that captures one of the previously defined variables indicating the relevant time periods for social media exposure, which may or may not vary by park. $1[\;\text{High}\;{\text{SME}}_{i}]$ indicates that site i is in the top half of the social media exposure distribution. Eq. **1** includes site-by-month fixed effects ($\alpha_{\mathrm{im}}$), which capture park-specific seasonal changes in visitation, year (y) fixed effects ($\lambda_{y}$), and the following controls: Monthly total precipitation and number of days maximum temperatures fall within 10-°C bins at the county-level, state-level real per capita income, and state-level unemployment rates. With year fixed effects, ${\hat{\beta }}_{1}$ is only identified when using post periods that vary by park. This approach can be interpreted similarly to a differences-in-differences strategy in which treatment is assigned to park sites with high social media exposure, although low SME parks may be affected by exposure as well and I explore those effects directly. Eq. **1** is estimated on a sample of visitation data from 2000 to 2019 for all 62 NPS sites. SEs are two-way clustered at the park-by-month and year-by-month level.

The primary coefficient of interest in Eq. **1** is ${\hat{\beta }}_{2}$, which is the average change in inverse-hyperbolic sine–transformed monthly visitation at NPS sites with high social media exposure relative to the parks with low exposure. This coefficient can be easily scaled to an approximate percentage change as $\exp({\hat{\beta }}_{2})-1$ (31). Because of the inclusion of park-by-month fixed effects, this coefficient is identified off of variation in visitation within a park before and after the social media era, accounting for park-specific seasonal visitation patterns, for parks in the top half of $SME_{i}$ relative to parks in the bottom half of $SME_{i}$. For ${\hat{\beta }}_{2}$ to be interpreted as causal, social media exposure must be orthogonal to visitation rates after conditioning on month-specific unobserved attributes of each park annual fixed effects and other controls.

For the heterogeneity analysis in Fig. 2 and *SI Appendix*, Fig. S7, I estimate a variant of Eq. **1** that includes an interaction term between $1\left[\;\text{Post}\right]\times 1\left[\;\text{High}\;{\text{SME}}_{i}\right]$ and indicators for different park characteristics, often represented by whether the park is above/below the median of the characteristic of interest. All heterogeneous results are estimated relative to the average low SME park.

Even with park-by-month fixed effects, social media exposure could be correlated with time-varying unobserved qualities of each park, potentially making social media exposure endogenous. To overcome this concern, I adopt an instrumental variables strategy in which the potentially endogenous $1\left[\;{\text{Post}}_{\mathrm{it}}\right]\times 1\left[\;\text{High}\;{\text{SME}}_{i}\right]$ is instrumented with a metric that captures the relative online popularity of each park site in the pre-social media era. Specifically, I construct a rank of relative Google Trends online search intensity for each park for the 2004 to 2009 period. This metric captures, for example, the relative intensity by which internet users were Googling for images of Yosemite National Park or searching for hiking trail maps for Shenandoah National Park online prior to the social media era. I then define a variable equal to one if site i is in the top half of the Google Trends rank and I use this variable, interacted with the post-period indicator variable, as an instrument for high social media exposure. By definition, the Google Trends rank does not influence visitation in the post period directly but is correlated with social media exposure after conditioning on unobserved time-invariant factors of each park. This relationship is illustrated on the right-hand side of *SI Appendix*, Fig. S9 and visitation trends for parks in the top/bottom half of this distribution are presented in *SI Appendix*, Fig. S8.

I also estimate Eq. **1** using annual park revenue as the dependent variable. The regression sample spans 2004 through 2019 due to data availability. Because revenue is only available at the annual level, I use park ($\alpha_{i}$) and year ($\lambda_{y}$) fixed effects, with SEs two-way clustered at the same level. I estimate this regression in OLS and IV frameworks identical to the visitation frameworks.

Further, I implement a different framework to estimate the dynamic effect of social media exposure on National Park visitation by leveraging Twitter users’ posts about individual National Parks in the preceding 12 mo. This strategy avoids the potentially endogenous definition of SME_*i*_ by relying on park-specific user-generated tweets in the preceding year as an alternative source of heightened social media exposure. I specify the following equation:

[2] $$\begin{array}{c} sinh^{-1}(V_{ismt})=\gamma Tweets\_12m_{it}+\gamma_{j}Tweets\_12m_{it}\times Tweets\_12m_{it}^{\;j}\\ +\;{\text{controls}}_{ist}+\alpha_{im}+\lambda_{y}+\varepsilon_{ismt}. \end{array}$$

Similar to the above, the dependent variable is the inverse hyperbolic sine of monthly visits at each park site. $Tweets\_12m_{\mathrm{it}}$ is a standardized, 12-mo rolling average of the number of park-specific tweets posted by public Twitter users in the previous year. $Tweets\_12m_{\mathrm{it}}^{\;j}$ is a standardized measure of j tweet characteristics posted by Twitter users, including the number of original tweets (mean: 685; SD: 2282), the number of retweets (mean: 1324; SD: 5616), the number of liked tweets (mean: 3604; SD: 13,665), the number of tweets with media attached (mean: 193; SD: 584), and the number of negative tweets (mean: 70; SD: 313). Each of these j tweet characteristics is interacted with the number of original tweets to show how different tweet attributes scale up or down the baseline effect of tweet quantities as an agnostic measure of social media exposure. Regression controls are the same as in Eq. **1**. The coefficient of interest is $\hat{\gamma }$ (or ${\hat{\gamma }}_{j}$), which captures how a SD increase in the quantity of tweets (with characteristic j) in the previous year influences visitation for a given park. Conditional on economic and weather controls and park-by-month and year fixed effects, user tweets about a park in year y−1 are exogenous to park-specific visitation in year y.

## Supplementary Material

Appendix 01 (PDF)

## Data Availability

Processed data and code to replicate all results in the analysis and *SI Appendix* are available at http://10.5281/zenodo.10444736 (39).

## Footnotes

^*^This analysis focuses on 62 NPS sites with the National Park designation and not other NPS designations, such as National Historic Sites, National Monuments, National Memorials, and so forth. There are currently 63 National Parks. New River Gorge National Park and Preserve was given the National Park designation in 2020; prior to 2020, it was a National River. As a result, this park is excluded from the analysis. See *SI Appendix*, Table S1 for a full list of parks included.

^†^Coefficients are changes in visitation transformed by the inverse hyperbolic sine. Ref. 31 shows that coefficients from $sinh^{-1}$–linear regressions with dummy variables can be interpreted as approximate percentage changes by transforming the estimated coefficient by $\exp(\hat{\beta })-1$.

^‡^However, the primary results suggest that the divergence in visitation between high and low SME parks begins in 2013, several years prior to the Centennial.

^§^Twitter’s public API allows a user to obtain a maximum of the 3,200 most recent tweets per account, which was binding for 13 National Parks.

^¶^In 2008, users sent 100 million tweets per quarter; in February 2010, users sent 50 million tweets per day. Source: https://www.businessofapps.com/data/twitter-statistics/.

^#^In 2013, Instagram had 110 million users, growing to 500 million in 2016. Source: https://www.businessofapps.com/data/instagram-statistics/.
