# Supplementary material for: Social media influences National Park visitation
Source: Proc Natl Acad Sci U S A. 2024 Apr 1;121(15):e2310417121. doi: 10.1073/pnas.2310417121 (PMC11009645; doi:10.1073/pnas.2310417121)
Supplement: Supplementary file 1 — Appendix 01 (PDF) [file pnas.2310417121.sapp.pdf]

Supporting Information for:
Social media influences National Park visitation

Corresponding author: Casey J. Wichman
Email: wichman@gatech.edu

This PDF file includes:

SI Text
Tables SI1 to SI4
Figures SI1 to SI10

SI.1 Supporting text

This section summarizes the supporting information presented in the subsequent tables and figures below.

Table SI1 presents each National Park analyzed. For each park, annual average recreational visits are presented for the 2005–2009 period, which is the pre-social media era. The percentage change in annual average recreational visits from 2005–2009 to 2015–2019 is also presented. The next two columns present the rank of each park's social media exposure (SME) and Google Trends (GT) index.

Table SI2 summarizes annual changes in visitation in the post period relative to the pre-social media era. Panel A presents OLS estimates for the inverse-hyperbolic sine of monthly visits at each park. Panel B presents fixed-effect Poisson estimates for monthly visits at each park. Each coefficient presents an average change in visitation in the post period relative to the pre-social media era, for each of the four post periods, with and without park-specific controls. None of the regressions include year fixed effects, so these estimates should be interpreted as raw changes in visitation.

Table SI3 illustrates a random sample of user tweets that contain the name of each park along with some summary statistics associated with the tweet, such as the Vader sentiment score, number of retweets, number of replies, number of likes, and whether media is attached. Any identifying information is removed.

Table SI4 presents estimates of the effect of social media exposure on visitation. Panel A presents OLS estimates for the natural log of monthly visits at each park. Panel B presents IV estimates for the natural log of monthly visits at each park. Panel C presents fixed-effect Poisson estimates for monthly visits at each park. Each coefficient presents an average change in visitation in the post period relative to the pre-social media era, for each of the four post periods, with and without park-specific controls. The results are generally in line with the primary results in Table 1. The IV estimates are slightly smaller than the OLS estimates, which is consistent with the IV estimates in Table 1. The Fixed-Effect Poisson estimates are generally larger than the OLS estimates.

Figure SI1 shows how visitation to National Parks varies with each component of the social media exposure index. The relationship generally mimics the trend shown in Figure 1, panel A, for the overall SME index.

Figure SI2 break down recreational visits and nonrecreational visits to National Parks by quartile of the social media exposure index. For recreational visits, the increase in the 2010s is generally increasing with the quartile of the social media exposure index. For nonrecreational visits, there is no visible change in vistation within each of the quartiles.

Figure SI3 presents the quarter of first posts for all National Park accounts on Twitter and Instagram as well as the aggregate number of posts on Twitter. These variables are useful for identifying the post periods in the primary analysis that relates changes in visitation to the SME index interacted with post-period indicators.

Figure SI4 presents the relationship between the social media exposure index and the Google Trends index. The relationship is positive and strong, suggesting that the social media exposure index is correlated with the Google Trends index. This relationship is used to instrument for social media exposure in the IV specifications in Table SI4.

Figure SI5 shows the estimates from an event study for changes in the inverse-hyperbolic sine of visitation before/after 2009 based on the OLS specification in Table 1 with park-by-month and year fixed effects and weather and economic controls. Panel A presents annual coefficients for High and Low SME parks separately, while Panel B presents the difference between the two coefficients. Panels C and D are the same specifications after removing two parks (Kobuk Valley NP and National Park of American Samoa) that had percentage changes in visitation exceeding 200% from 2005–2009 to 2015–2019.

Figure SI6 shows the total revenue generated by each National Park site over time broken out by parks in the high/low end of the social media exposure index (panel A). In panels B–D, I show the same trends for three main types of revenue generated within the park: entrance fees, annual pass sales, and recreation/camping fees. Revenue from entrance fees and annual pass sales follow the same pattern as visitation: they are largely flat, then increase sharply around 2014, with revenue at High SME parks increasing at a proportionally greater rate. For recreation/camping fees, the trends are similar for both High and Low SME parks, with a roughly flat trend over time and decreases in later years of the sample for both groups.

Figure SI7 illustrates heterogeneity in the effect of being a High SME park on visitation based on observable characteristics of the park. The figure is the same as Figure 2 in the main paper, but uses the SME index interacted with the post-Instagram indicator variable. The results are similar to the results in Figure 2.

Similar to the previous figures, Figure SI8 breaks down visitation by whether parks are in the top/bottom half of the Google search intensity rank. The relationship is similar to the relationship between visitation and the social media exposure index, emphasizing the validity of the instrument used in the IV specifications in Table 1.

Figure SI9 shows the relationship between parks' rank of social media exposure relative to the rank of deferred maintenance and repairs (DM&R) per visitor. The relationship is not particularly strong. There are some larger parks with high SME and low DM&R per visitor clustered in the top left. There are also some prominent parks (e.g., Yellowstone, Yosemite, and Grand Canyon) with high SME and high DM&R per visitor clustered in the bottom left. Smaller parks tend to fall in the bottom right with low ranks of SME and relatively high ranks of DM&R per visitor.

Finally, Figure SI10 presents a scatterplot of the relationship between different components of the social media exposure index. The relationship suggests that each of the components are in strong agreement with one another, suggesting a high degree of correlation between the different components of the social media exposure index. This figure implies that the social media exposure index is likely robust to exclusion of various characteristics.

Table SI1: Summary statistics for each National Park

	Rec. visits	Pct. change	SME rank	GT rank
Acadia NP	2,128,171	56.0	20	12
Arches NP	880,001	78.3	10	23
Badlands NP	883,114	13.7	7	21
Big Bend NP	357,715	18.2	28.5	15
Biscayne NP	562,763	-5.9	34	52
Black Canyon of the Gunnison NP	178,495	67.6	39	41
Bryce Canyon NP	1,036,124	130.8	16	14
Canyonlands NP	415,287	74.7	23	27
Capitol Reef NP	567,738	97.6	31	39
Carlsbad Caverns NP	412,747	13.3	35.5	22
Channel Islands NP	370,218	-0.1	42	51
Congaree NP	112,351	24.0	28.5	60
Crater Lake NP	433,474	61.9	35.5	13
Cuyahoga Valley NP	2,581,364	-12.7	37	50
Death Valley NP	789,837	81.4	8	25
Denali NP & PRES	413,623	44.4	11	17
Dry Tortugas NP	60,548	10.6	40	53
Everglades NP	997,125	-4.9	18	24
Gates of the Arctic NP & PRES	10,351	0.6	57	58
Gateway Arch NP	2,443,471	-30.9	55	48
Glacier Bay NP & PRES	413,739	39.6	30	30
Glacier NP	1,962,441	49.1	3	4
Grand Canyon NP	4,373,602	37.6	4	1
Grand Teton NP	2,504,912	32.8	12	8
Great Basin NP	78,368	82.2	44	44
Great Sand Dunes NP & PRES	277,446	54.6	60	36
Great Smoky Mountains NP	9,277,878	23.6	9	35
Guadalupe Mountains NP	174,448	7.5	49	42
Haleakala NP	1,303,548	-13.6	41	33
Hawaii Volcanoes NP	1,448,973	13.5	25	26
Hot Springs NP	1,287,324	16.5	50	49
Indiana Dunes NP	1,963,195	-4.4	46	31
Isle Royale NP	15,826	56.8	61	55
Joshua Tree NP	1,325,486	100.9	6	7
Katmai NP & PRES	51,309	-8.2	21	38
Kenai Fjords NP	257,016	26.5	32	47
Kings Canyon NP	582,391	6.4	47	28
Kobuk Valley NP	2,467	400.3	62	61
Lake Clark NP & PRES	6,558	184.6	27	57
Lassen Volcanic NP	378,467	33.6	33	34
Mammoth Cave NP	782,655	-27.8	48	16
Mesa Verde NP	539,701	6.1	58	20
Mount Rainier NP	1,130,013	24.4	13.5	5
National Park of American Samoa	3,734	975.8	59	62
North Cascades NP	20,617	43.5	38	37
Olympic NP	3,047,713	7.7	22	18
Petrified Forest NP	583,795	14.9	24	32
Pinnacles NP	165,787	27.2	43	40
Redwood NP	400,884	24.5	17	19
Rocky Mountain NP	2,803,428	59.6	13.5	11
Saguaro NP	674,008	34.0	26	43
Sequoia NP	966,814	26.6	15	10
Shenandoah NP	1,095,030	26.2	19	9
Theodore Roosevelt NP	497,775	39.9	45	45
Virgin Islands NP	587,318	-52.3	52	59
Voyageurs NP	226,223	5.2	53	54
White Sands NP	447,619	28.6	54	29
Wind Cave NP	593,597	5.2	51	46
Wrangell-St. Elias NP & PRES	58,661	30.1	56	56
Yellowstone NP	3,043,811	35.4	2	3
Yosemite NP	3,443,840	27.5	1	2
Zion NP	2,647,370	60.6	5	6

Notes: "Rec. visits" is annual average recreational visitation in 2005–2009. "Pct. change" is percentage change ($\times$100) in annual average recreational visitation from 2005–2009 to 2015–2019. "SME rank" is rank of social media exposure (half percentages indicate ties). "GT Rank" is rank of Google trends index (half percentages indicate ties).

Table SI2: Average increase in visitation in post period

Panel A: OLS estimates for $\sinh^{-1}$(visits)

	Post-2010		Post-2015		Post Twitter		Post Instagram	
	(1)	(2)	(3)	(4)	(5)	(6)	(7)	(8)
$1[\text{Post } 2010_t]$	0.12^{***}	0.06^{**}						
	(0.02)	(0.02)						
$1[\text{Post } 2015_t]$			0.25^{***}	0.23^{***}				
			(0.02)	(0.03)				
$1[\text{Post Twitter}_{it}]$					0.16^{***}	0.08^{***}		
					(0.02)	(0.02)		
$1[\text{Post Instagram}_{it}]$							0.26^{***}	0.23^{***}
							(0.02)	(0.03)
Obs.	14,831	14,141	14,831	14,141	14,831	14,141	14,831	14,141
Adj. R2	0.92	0.93	0.92	0.93	0.92	0.93	0.92	0.93
Park-by-month FEs?	Y	Y	Y	Y	Y	Y	Y	Y
Controls?	–	Y	–	Y	–	Y	–	Y

Panel B: Fixed-effect Poisson estimates for recreational visits (in levels)

	Post-2010		Post-2015		Post Twitter		Post Instagram	
	(1)	(2)	(3)	(4)	(5)	(6)	(7)	(8)
RecreationVisits								
$1[\text{Post } 2010_t]$	0.12^{***}	0.06^{***}						
	(0.01)	(0.01)						
$1[\text{Post } 2015_t]$			0.22^{***}	0.16^{***}				
			(0.01)	(0.01)				
$1[\text{Post Twitter}_{it}]$					0.17^{***}	0.08^{***}		
					(0.01)	(0.01)		
$1[\text{Post Instagram}_{it}]$							0.22^{***}	0.15^{***}
							(0.02)	(0.02)
Obs.	14,831	14,141	14,831	14,141	14,831	14,141	14,831	14,141
Adj. R2								
Park-by-month FEs?	Y	Y	Y	Y	Y	Y	Y	Y
Controls?	–	Y	–	Y	–	Y	–	Y

Notes: Dependent variable is inverse-hyperbolic sine of monthly recreation visits at National Park sites. Controls include county-level temperature and precipitation, state-level real per capita income, and state-level unemployment rate. Standard errors are two-way clustered at the park-by-month and year-by-month level in Panel A. Robust standard errors are presented in Panel B. *, **, and *** represents signficance at the $p < 0.1$, $p < 0.05$, and $p < 0.01$ level.

Table SI3: Example text from user tweets containing park names

text	vader score	retweets	replies	likes	media attached
@glaciernps and @yellowstonenps attract most of #montana's tourists and spending according to a new report	0.361	0	0	0	1
@joshuatreenps haha, true that! best way to experience california for sure!	0.917	0	0	0	0
#anseladams in space! mt @interior this photo @grandtetonnps is heading out of the #solarsystem on @nasavoyager	0.000	1	0	0	0
a9: cross-country skiing is a great way to access #exitglacier while the road is closed. 16 mi rt to view the glacier. ...	0.625	4	1	14	1
@interior @archesnps social media marketing #findnerd #seo #web #mobileappdevelopment	0.000	0	0	1	1
#sierranevada #yosemite #nature #greatoutdoors	0.000	0	0	0	0
cnn: a tree limb fell on a tent in yosemitenps early friday, killing two youths who were sleeping inside, the park said. ...	-0.660	0	0	0	0
new snow. roads are snow-covered; plows working. ks	-0.153	1	0	4	1
@yellowstonenps yes! we were very lucky to see them. being at yellowstone was one of the best experiences of my life!	0.890	0	0	3	0
@greatsmokynps @natlparkservice that's a great drive. i love that portion of gsmnp #roadtripchat	0.852	0	0	0	0
unfortunately, i see a lot of dog owners in the #shenandoahnps run their dogs off leash.	-0.340	0	1	1	0
4 a truly enchnated magical holiday head 4 @greatsmokynps	0.681	0	0	0	0
please let someone offer a job to the person about to get fired from @badlandsnps. we love you!!! #gogogo	0.671	0	0	2	0
@yosemitenps yosemite inspired me to submit a concept design of john muir admiring yosemite's beauty. #yosemite150	0.862	0	0	0	1
oh yes you guys are - been to waterton twice and loved it. its a must go place	0.765	0	0	1	0
haha yes we noticed that!!! @yosemitenps	0.763	0	0	0	0
today's adventure takes us to badlands national park in south dakota, usa. #nationalparks #hiking #travel @badlandsnps	0.318	6	2	9	1
thousands of daring hunters descend on @evergladesnps for #pythonchallenge:	0.361	1	0	1	1
@grandcanyonnps	0.000	0	0	0	0
this #bull #elk stopping for a drink of water while i was watching it. #yellowstonenps #wyoming #wildlife #animals...	-0.153	0	0	0	0
@ncascadesnps my favorite road in washington! thanks for all the hard work! be safe!	0.851	0	0	1	0
my favorite pic i snapped today @rockynps: elk at horseshoe park. and i did it quick – it was like 5 degrees. #9wx	0.670	7	0	31	1
bison and clouds move slowly across the landscape during a beautiful sunrise in lamar valley (via @yellowstonenps)	0.599	1	0	2	1
another record breaking year for .@greatsmokynps!	0.000	0	0	3	1
@rockynps @natlparkservice sweet! we haven't been back w kids, and in fact will also be there in june for a week! i have heard...	0.862	0	3	3	0
@yosemitenps it is! we can't wait to go back!!	0.000	0	0	0	0
@interior @deathvalleynps amazing photo. thanks for sharing.	0.859	0	0	0	0
checking out some of the horse to remember to cowboy past. . photo credit: at @capitolreefnps use #nationalparkobsessed...	0.557	0	0	0	1
the damaged pipeline that supplies water to the north rim of @grandcanyonnps has been repaired, and most water services...	-0.128	0	0	4	0
full of great geological features that'll keep you star-eyed zion's well known for its great mountains! @zionnps	0.891	0	0	0	1
beautiful.	0.599	0	0	1	0
@joshuatreenps thank you!	0.420	0	0	0	0
just over 2 years ago, i went to @katmainps on the wildest day trip of my life, but in the end was rewarded. #brownbear	0.649	1	0	5	1
@acadianps we agree! this is a gorgeous pic of a beautiful place.	0.893	0	0	0	0
a perfect birthday hike to wheeler peak for @greatbasinnps #findyourpark	0.572	0	0	1	1
wow, the thought: bats yelling outside my window at night....because they are here: little browns that roost in my barn.	0.586	0	0	2	0
@grandcanyonnps @natlparkservice please tell @potus to preserve all of our parks exactly as they are and not sell parts off to...	0.758	0	0	0	0
teddy roosevelt	0.000	0	0	0	0
@gatesarcticnps p'rafted the alatna last summer and backpacked to the arrigetch. we did not spend enough time in the mount...	0.223	0	1	2	1

Notes: Random sample of tweets from the universe of tweets that contain the NPS site's Twitter handle (e.g., "@YosemiteNPS"). Vader score is the sentiment score from the VADER sentiment analysis algorithm; positive values indicate positive sentiment, negative values indicate negative sentiment. Retweets, replies, and likes are counts of the number of times the given tweet was retweeted, replied to, or liked. Media attached is a binary indicator for whether the tweet contains a photo or video. Any identifying information (i.e., personal Twitter handles) have been removed. Emojis have also been removed. Some tweets have been truncated.

Table SI4: Effect of social media exposure (SME) on National Park visitation with logged outcomes as well as fixed-effect poisson regressions

**Panel A:** OLS estimates for ln(visits)				
	Post 2010	Post 2015	Post Twitter	Post Instagram
1[Post]			-0.19***	-0.10***
			(0.04)	(0.03)
1[Post] × 1[High SME$_i$]	0.20***	0.22***	0.28***	0.23***
	(0.03)	(0.03)	(0.03)	(0.03)
Obs.	14,031	14,031	14,031	14,031
R2 (adj.)	0.97	0.97	0.97	0.97

**Panel B:** IV estimates for ln(visits)				
	Post 2010	Post 2015	Post Twitter	Post Instagram
1[Post]			-0.12***	-0.08**
			(0.04)	(0.04)
1[Post] × 1[$\widehat{\text{High SME}}_i$]	0.08	0.14***	0.16***	0.19***
	(0.05)	(0.05)	(0.04)	(0.05)
Obs.	14,031	14,031	14,031	14,031
R2 (centered)	0.03	0.04	0.04	0.04
Kleibergen-Paap F statistic	375.3	394.8	402.1	542.4

**Panel C:** Fixed-effect Poisson estimates for recreational visits in levels				
	Post 2010	Post 2015	Post Twitter	Post Instagram
RecreationVisits				
1[Post]			-0.27***	-0.20***
			(0.03)	(0.04)
1[Post] × 1[High SME$_i$]	0.31***	0.32***	0.38***	0.33***
	(0.03)	(0.03)	(0.03)	(0.04)
Obs.	14,141	14,141	14,141	14,141
Log Pseudolikelihood	-2.61e+07	-2.66e+07	-2.58e+07	-2.67e+07

Notes: Panels A and B: Dependent variable is the natural log of monthly recreation visits at National Park sites. Each column reflects a different definition of 1[Post]: "Post 2010" equals one for all time periods $\geq$ 2010; "Post 2015" equals one for all time periods $\geq$ 2015; "Post Twitter" equals one for all time periods after the park's first tweet; "Post Instagram" equals one for all time periods after the park created its Instagram account. All specifications include county-level temperature and precipitation, state-level real per capita income, state-level unemployment rate, park-by-month fixed effects, and year fixed effects. Standard errors are two-way clustered at the park-by-month and year-by-month level in Panels A and B. Robust standard errors are presented in Panel C. *, **, and *** represents signficance at the $p < 0.1$, $p < 0.05$, and $p < 0.01$ level.

Figure SI1: Visits over time by individual components of SME, 1980–2019

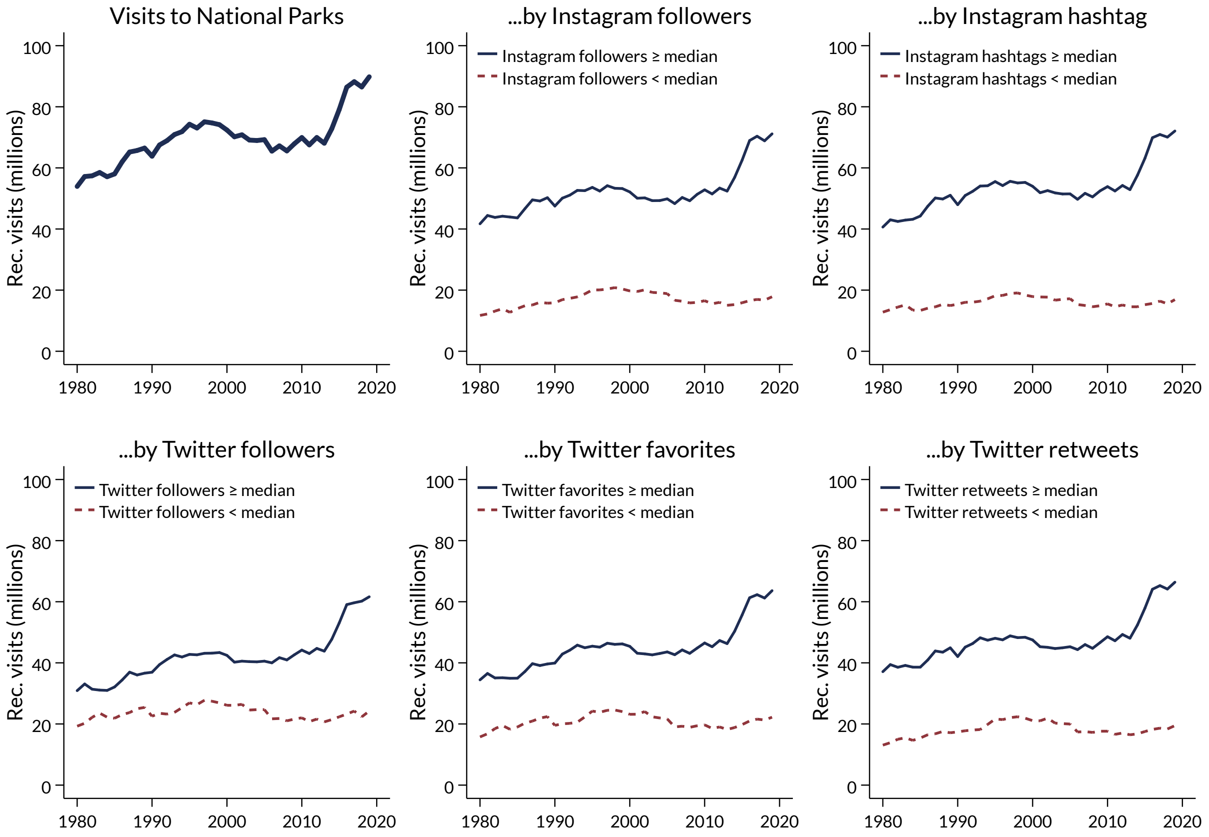

Notes: This figure presents total annual recreational visits to all National Parks and disaggregated by parks in top/bottom half of component of the Social Media Exposure index. Top left: total recreational visits over time for all parks. Top center: total recreational visits over time for parks disaggregated by Instagram followers (i.e., above/below median). Top right: total recreational visits over time for parks disaggregated by Instagram hashtags (i.e., above/below median). Bottom left: total recreational visits over time for parks disaggregated by Twitter follows (i.e., above/below median). Bottom center: total recreational visits over time for parks disaggregated by Twitter favorites (i.e., above/below median). Bottom right: total recreational visits over time for parks disaggregated by Twitter retweets (i.e., above/below median).

Figure SI2: Recreational and non-recreational visits over time by Social Media Exposure rank quartile, 1980–2019

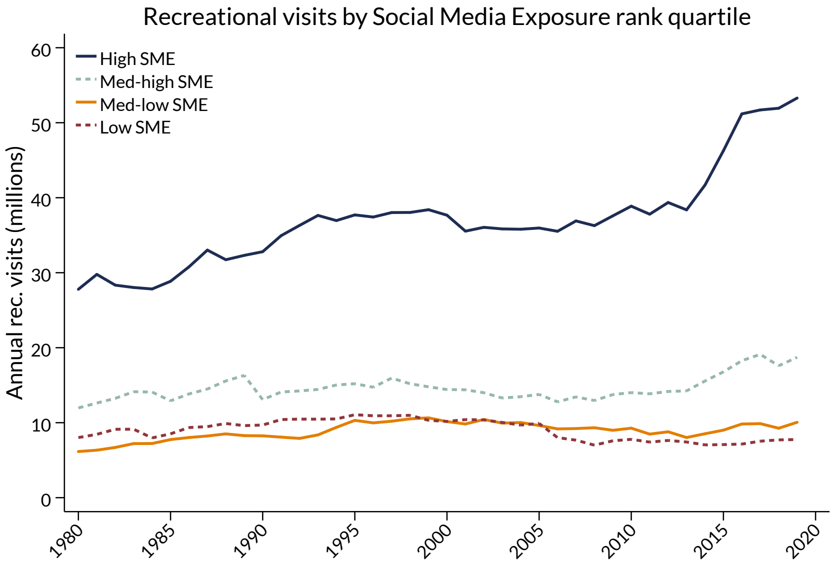

(a) Visits over time by Social Media Exposure rank quartile, 1980–2019

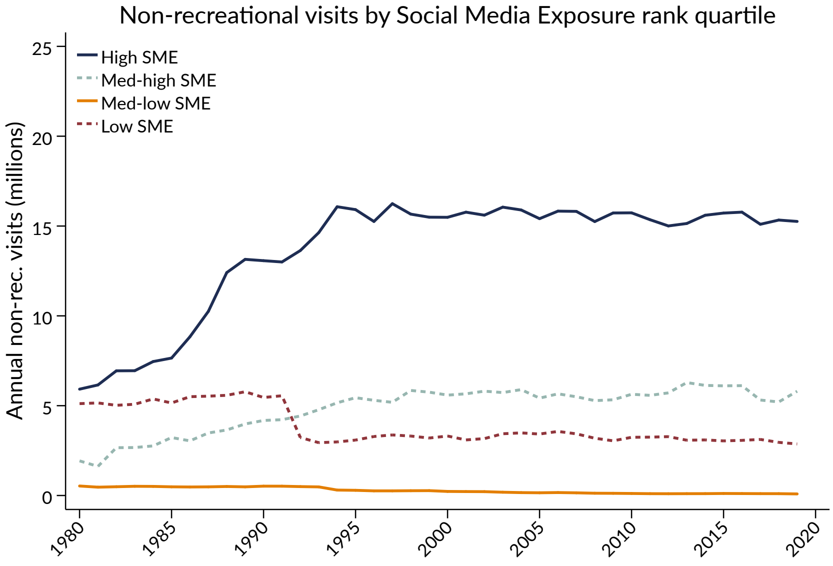

(b) Non-recreational visits over time by Social Media Exposure rank, 1980–2019

Notes: Panel A presents total annual recreational visits to all National Parks disaggregrated by quartiles of the social media exposure index. Panel B presents total annual nonrecreational visits (e.g., visits from guides, contractors, commuters on NPS roads, outside researchers) to all National Parks disaggregrated by quartiles of the social media exposure index. High SME = top quartile of SME index; Med-high SME = third quartile of SME index; Med-low = second quartile of SME index; Low SME = bottom quartile of SME index.

Figure SI3: Date of first social media post for National Parks and number of tweets per quarter for all parks.

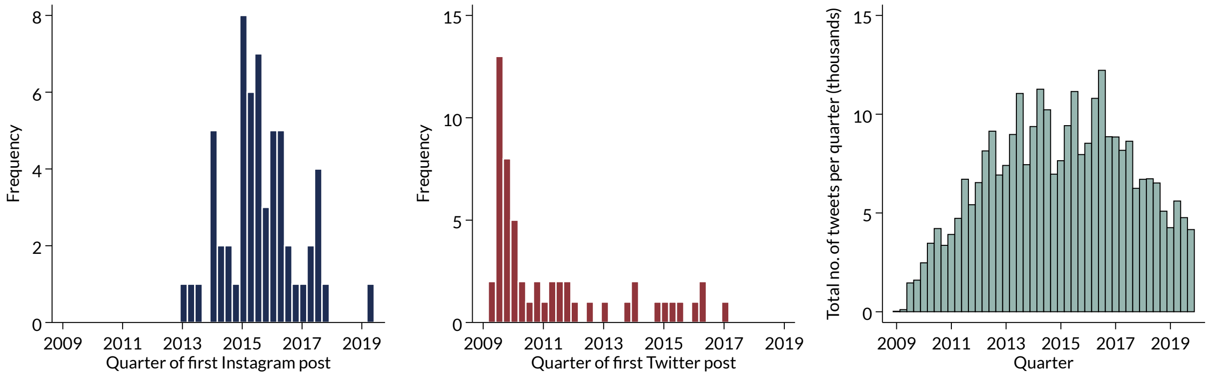

Notes: Left panel: Quarter of first instagram post for each park. Central panel: Quarter of first Twitter post for each park. Right panel: Total number of outgoing tweets in each quarter for all parks combined.

Figure SI4: Social media exposure relative to baseline Google search intensity (2004–2009)

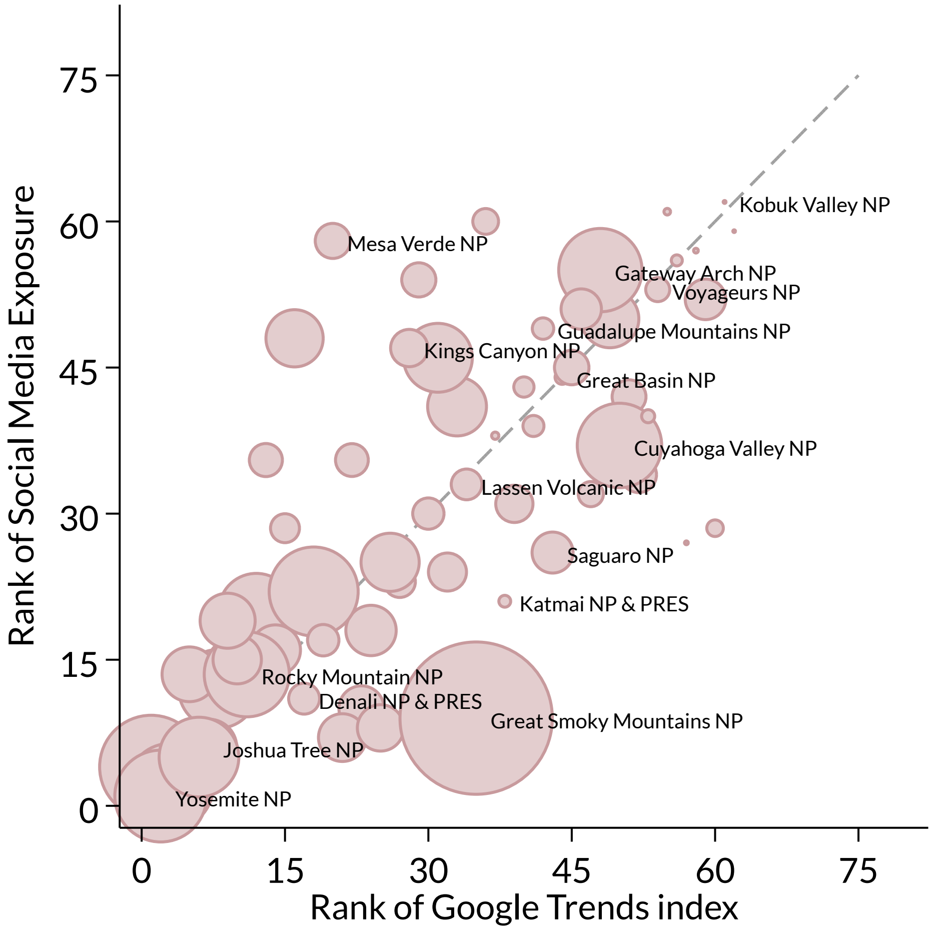

Notes: This figure presents the correlation between the rank of social media exposure and the rank of the Google Trends index for internet searches between 2004 and 2009. Size of circle indicates relative size of mean annual visitation for 2000–2009 for each NPS site.

Figure SI5: Event study for monthly visitation relative to 2009

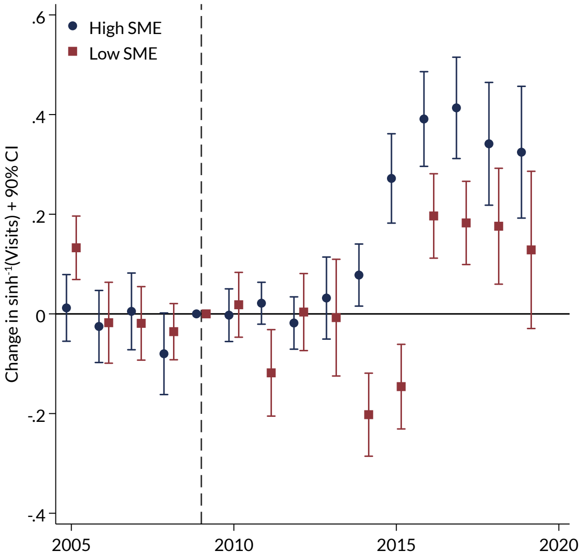

(a) Annual coefficients for parks with high and low SME

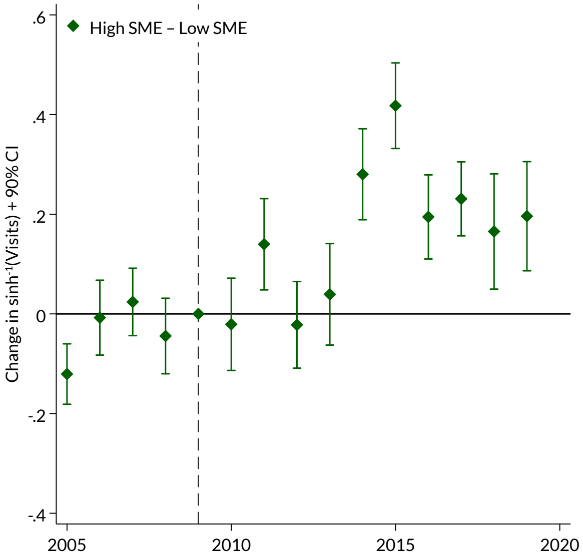

(b) Annual coefficients for difference between high and low SME parks

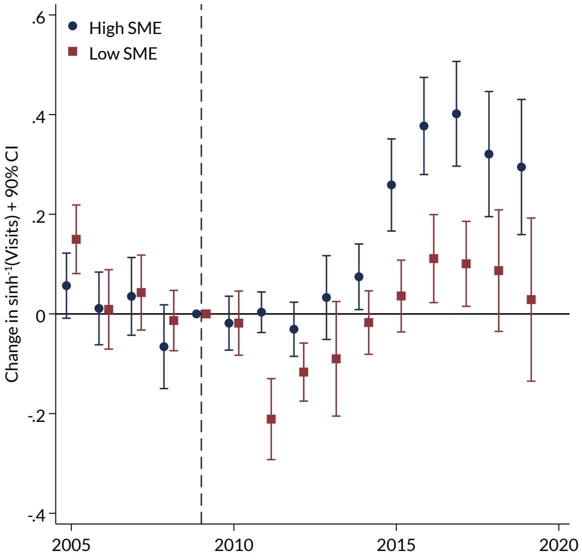

(c) Annual coefficients for parks with high and low SME - removing parks with absolute % change in visitation > 200% from 2005-2009 to 2015-2019

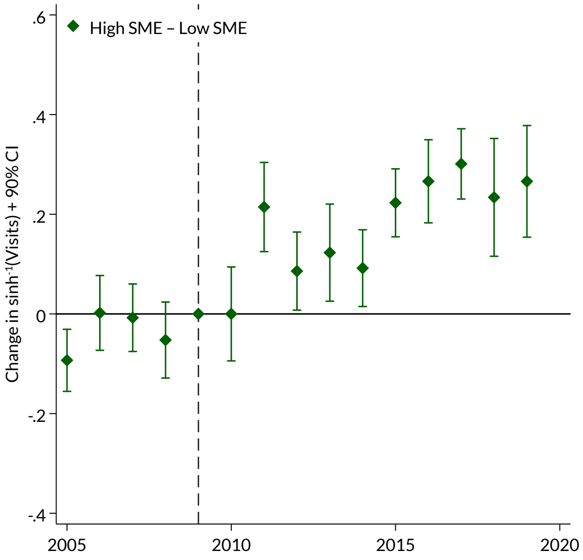

(d) Annual coefficients for difference between high and low SME parks - removing parks with absolute % change in visitation > 200% from 2005-2009 to 2015-2019

Notes: Panel A: Coefficients represent dummy variables for each year relative to 2009 interacted with 1[High SME] and 1[Low SME]. Regressions include weather/economic controls and park-by-month fixed effects. Sample is limited to 2005–2019. Panel B: Coefficients represent dummy variables for each year relative to 2009 interacted with 1[High SME]. Regression includes weather/economic controls park-by-month and year fixed effects. Dependent variable is the inverse-hyperbolic sine of monthly recreational visits at each National Park. Panels C and D are the same specifications as in Panels A and B, but after removing two parks (Kobuk Valley NP and National Park of American Samoa) that had percentage changes in visitation exceeding 200% from 2005–2009 to 2015–2019.

Figure SI6: Annual park revenue by social media exposure

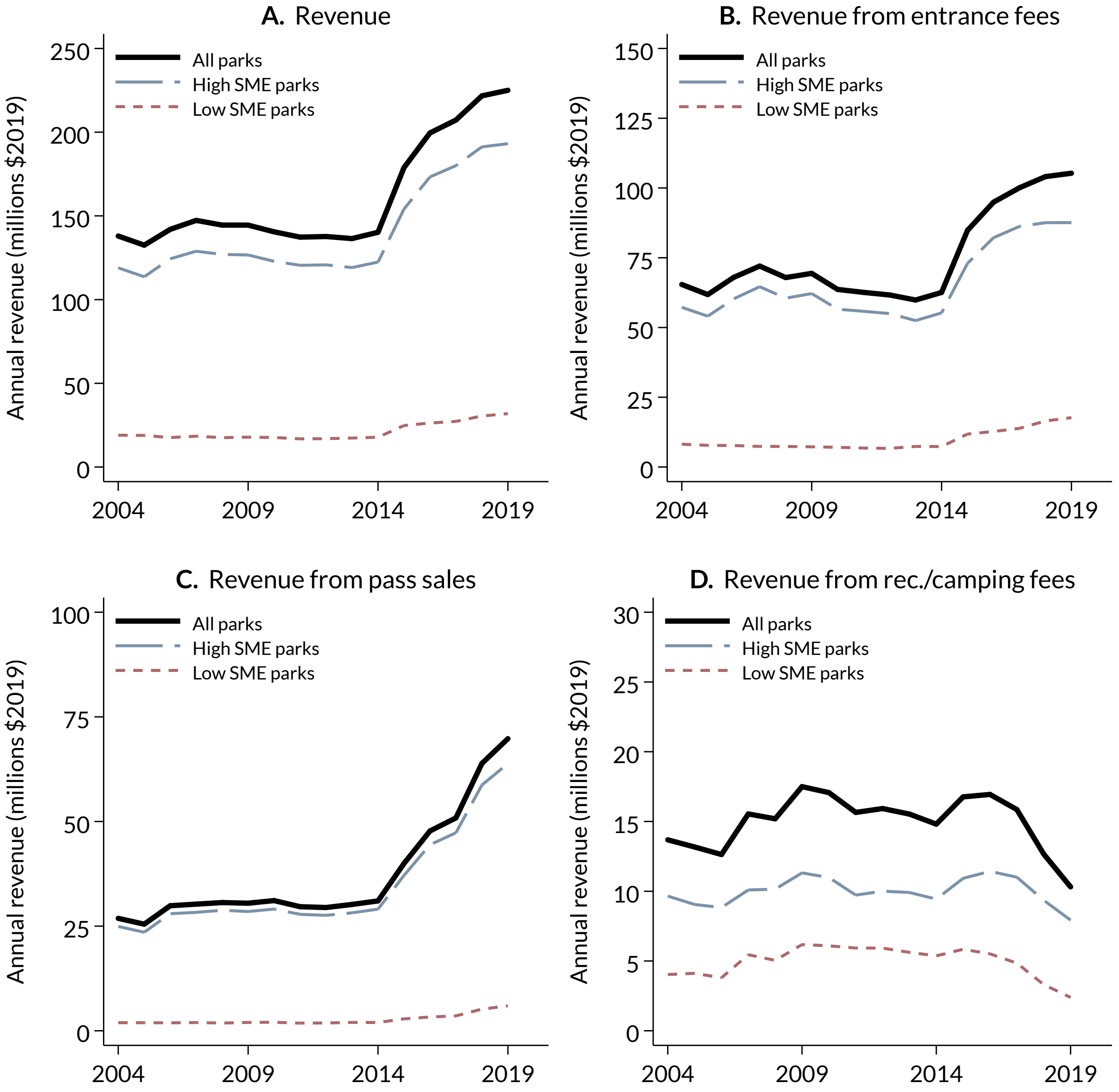

Notes: Panel A presents total annual revenue raised within all National Parks and disaggregated by parks in the bottom/top half of the Social Media Exposure index. Panel B is the same as Panel A, but for revenue raised through park entrance fees. Panel C is the same as Panel A, but for revenue raised from pass sales within the park (e.g., annual passes, senior passes, etc.). Panel D is the same as Panel A, but for revenue raised from recreation permits and fees (e.g., backcountry permits, camping fees, etc.).

Figure SI7: Heterogeneity in SME effect on visitation by park characteristics

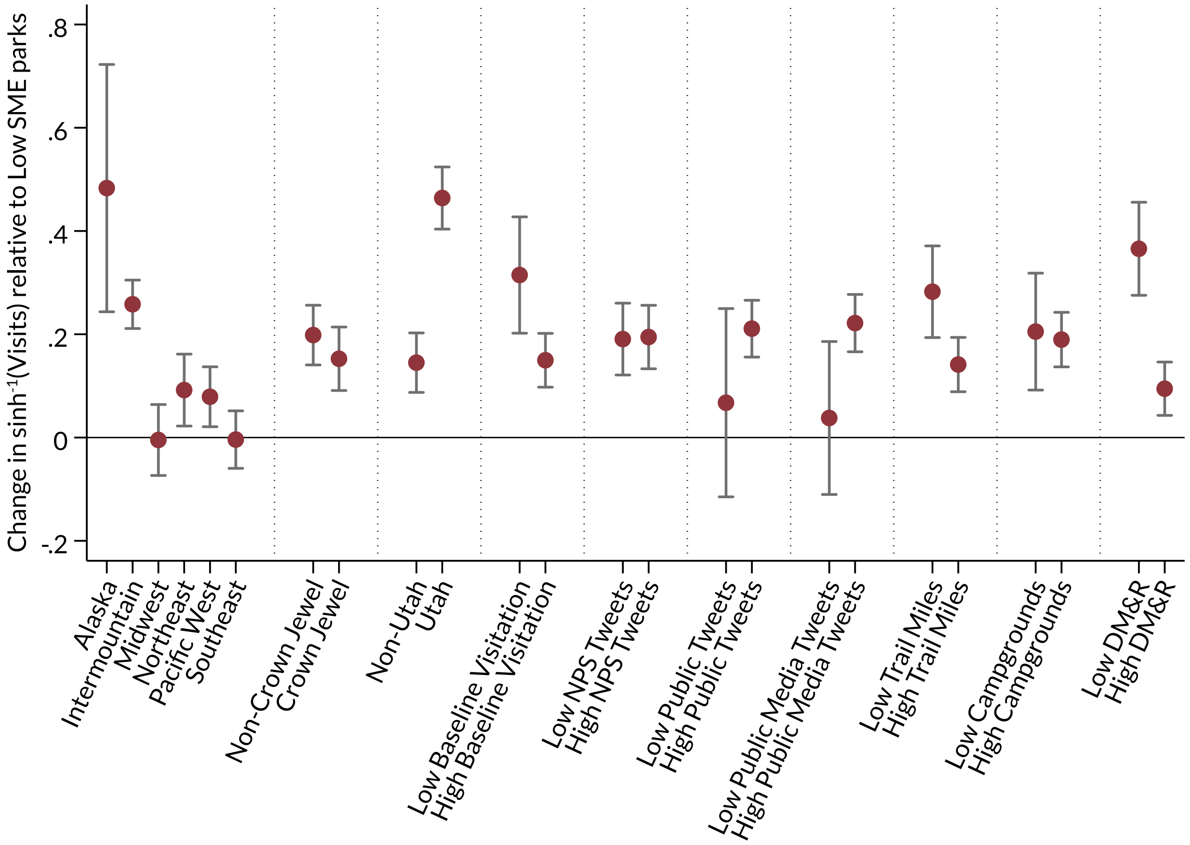

Notes: Each set of coefficients represents estimates from ten different regression specifications where the heterogeneous effects are identified via interactions with 1[Post Instagram$_{it}$] $\times$ 1[High SME$_i$]. Figure shows results for Post 2015. Coefficients represent changes in monthly visitation (transformed via the inverse hyperbolic sine) for High SME parks relative to the average Low SME park. 90% confidence intervals are shown in brackets. All regressions include park-by-month and year fixed effects and weather and economic controls. First set of results displays regional heterogeneity. Crown Jewel equals 1 for Yosemite NP, Yellowstone NP, and Grand Canyon NP. Utah equals 1 for any park in Utah. High/low baseline visitation is whether park is above/below median visitation in 2000–2009. High/low NPS tweets is whether park is above/below median number of tweets from officail NPS account in 2000–2019. High/low public tweets is whether park is above/below median number of tweets from non-NPS accounts in 2000–2019. High/low Public Media Tweets is whether park is above/below median number of tweets from non-NPS accounts with media attached in 2000–2019. High/low trail miles is whether park is above/below median number of hiking trail miles within park. High/low campgrounds is whether park is above/below median number of campgrounds within park. High/low deferred maintenance is whether park is above/below median deferred maintenance in FY2022.

Figure SI8: Recreational visitation by high/low Google search intensity

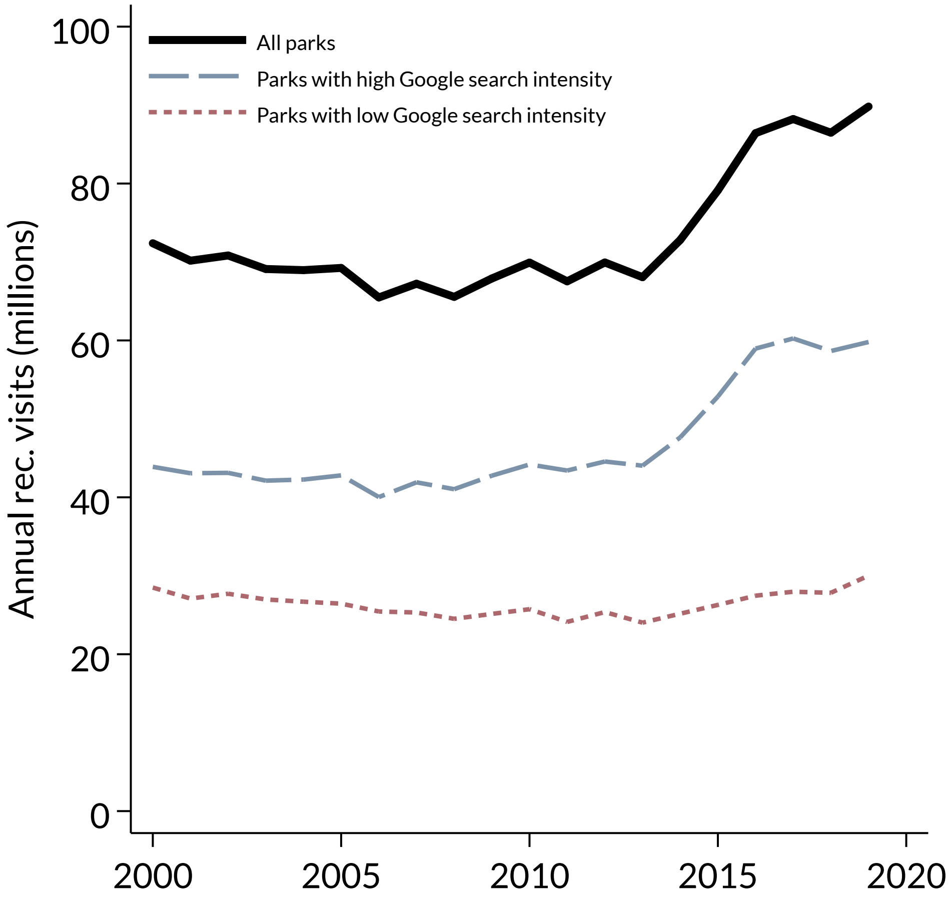

Notes: Annual recreational visits to all National Parks and disaggregated by parks in top/bottom half of relative Google Trends index distribution. The Google Trends index is the relative search intensity for variants of the park's name between 2004 and 2009.

Figure SI9: Scatter plot of rank of Social Media Exposure vs. rank of deferred maintenance & repairs per visitor

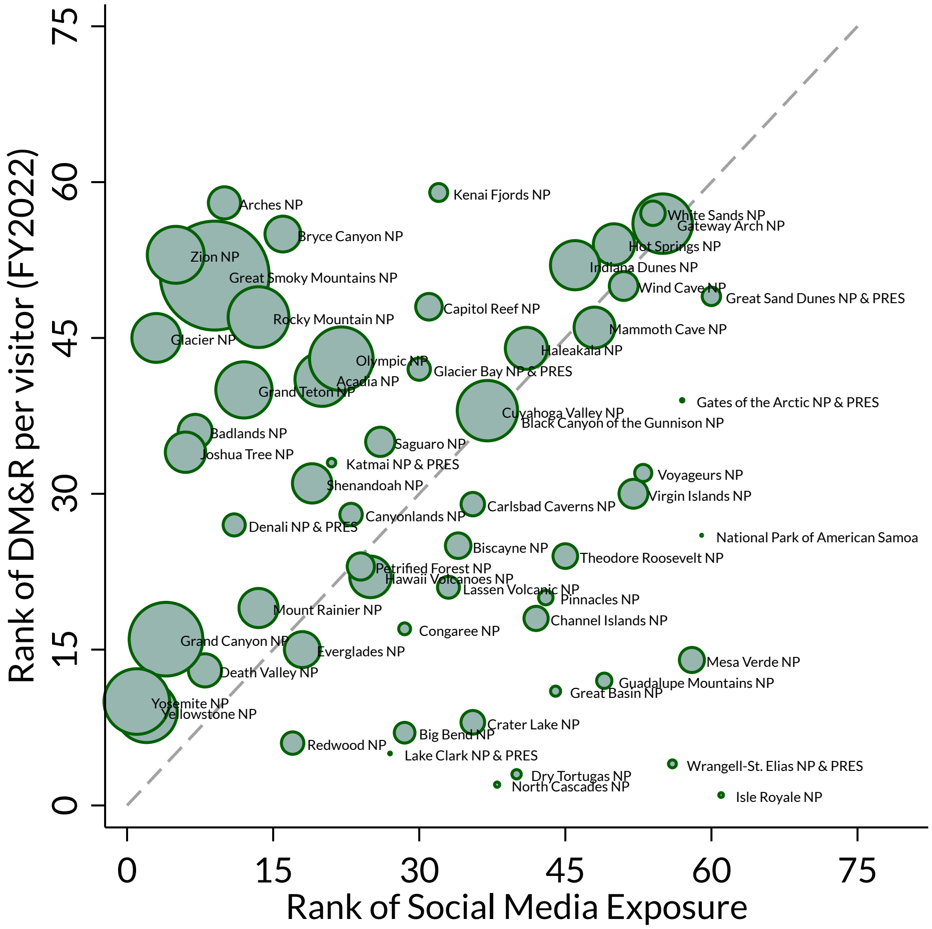

Notes: Size of circle indicates relative size of mean annual visitation for 2000–2009 for each NPS site. Deferred maintenance & repairs (DM&R) is FY2022 data divided by number of visitors in 2000–2009.

Figure SI10: Correlations across components of social media exposure index

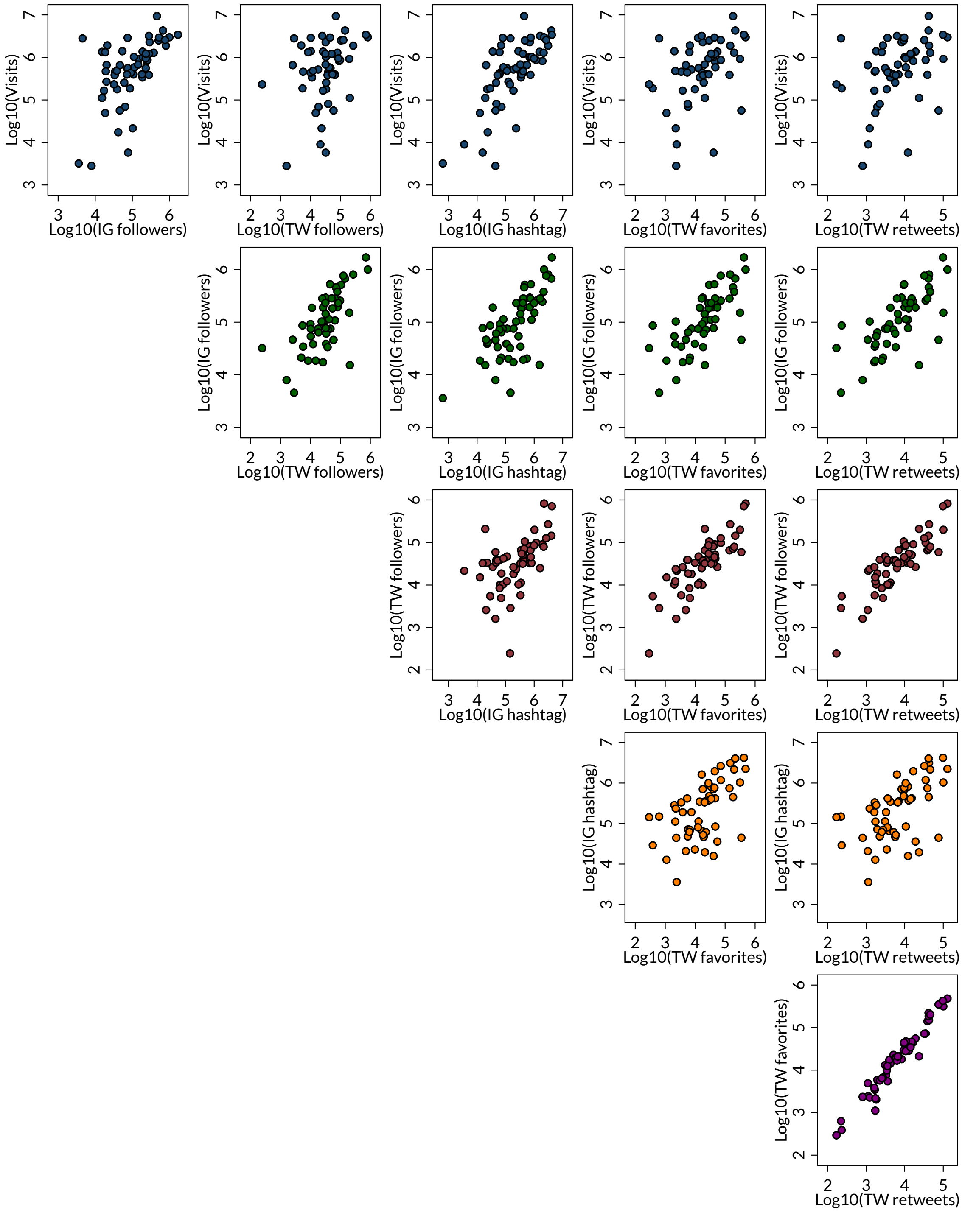

Notes: This figure presents a correlation matrix for each of the components of the social media exposure index relative to the average number of recreational visits at each park between 2000 and 2009. The SME components include: number of Instagram followers, number of Twitter followers, number of Instagram hashtags, number of Twitter favorites, and number of Twitter retweets. All variables are transformed by logarithms with base 10.
